# Towards quantitative root hydraulic phenotyping: novel mathematical functions to calculate plant-scale hydraulic parameters from root system functional and structural traits

**DOI:** 10.1007/s00285-017-1111-z

**Published:** 2017-03-02

**Authors:** F. Meunier, V. Couvreur, X. Draye, J. Vanderborght, M. Javaux

**Affiliations:** 1 Earth and Life Institute-Environment, Université catholique de Louvain, Louvain-la-Neuve, Belgium; 2 Department of Land, Air and Water Resources, University of California, Davis, CA USA; 3 Earth and Life Institute-Agronomy, Université catholique de Louvain, Louvain-la-Neuve, Belgium; 4 Forschungszentrum Jülich GmbH, Agrosphere (IBG-3), Jülich, Germany; 5 Division of Soil and Water Management, KU Leuven, Leuven, Belgium

**Keywords:** Root water uptake, Plant-scale parameters, Hydraulic architecture, Water flow equation in root, 92C80, 92B05

## Abstract

Predicting root water uptake and plant transpiration is crucial for managing plant irrigation and developing drought-tolerant root system ideotypes (i.e. ideal root systems). Today, three-dimensional structural functional models exist, which allows solving the water flow equation in the soil and in the root systems under transient conditions and in heterogeneous soils. Yet, these models rely on the full representation of the three-dimensional distribution of the root hydraulic properties, which is not always easy to access. Recently, new models able to represent this complex system without the full knowledge of the plant 3D hydraulic architecture and with a limited number of parameters have been developed. However, the estimation of the macroscopic parameters a priori still requires a numerical model and the knowledge of the full three-dimensional hydraulic architecture. The objective of this study is to provide analytical mathematical models to estimate the values of these parameters as a function of local plant general features, like the distance between laterals, the number of primaries or the ratio of radial to axial root conductances. Such functions would allow one to characterize the behaviour of a root system (as characterized by its macroscopic parameters) directly from averaged plant root traits, thereby opening new possibilities for developing quantitative ideotypes, by linking plant scale parameters to mean functional or structural properties. With its simple form, the proposed model offers the chance to perform sensitivity and optimization analyses as presented in this study.

## Introduction

Plant transpiration is the process by which water molecules are evaporated from leaf surfaces. When stomatas open, carbon dioxide is taken up while water vapour is lost at a certain rate function of the atmospheric evaporative demand, leaf properties and stomatal aperture (Lobet et al. 2014).

Root systems (RS) play a key role by providing water from the soil, which sustains the transpiration flux (McElrone et al. 2013) and by sending hydraulic and hormonal signals to regulate stomata conductance (Tardieu et al. 2015). Location of roots within the soil, connection between root segments but also root and soil hydraulic resistance distribution determine the ability of a root system to acquire and transport water. Thus, not only the RS architecture (de Dorlodot et al. 2007) but also the RS hydraulics (Vadez 2014) influence the ability of plants to extract and transport soil water to the leaves. It has been shown that RS architecture and hydraulics are determinant under drought (Leitner et al. 2014b; Tardieu 2012). Therefore, researchers have attempted to search for ideal structural and functional characteristics of root systems, which would help plant RS to explore soil and to provide water to the plant in an optimal way (Wasson et al. 2012; Lynch 2013). High plant conductivity, steep secondary roots, long roots, high radial conductivity, low xylem have for instance been proposed as potential optimal features (Comas et al. 2013). However, these features may not be optimal under all climatic and environmental conditions (Tardieu 2012; Leitner et al. 2014b). In addition, quantitative approaches are missing to properly evaluate how these proposed features would affect the plant ability to extract water.

Since the first studies of Dixon and Joly (1895) more than a century ago, the understanding of the sap ascent in plant has evolved until the establishment of the cohesion-tension theory (Steudle 2001; Wheeler and Stroock 2008). The water transport in xylem vessels is a mainly passive process opposed to the potential hydraulic gradient between the soil and the atmosphere and the continuity of the water phase is ensured (as long as cavitation does not appear) by tension-cohesion forces. An old and widely accepted model for plant water flow uses the Ohm law analogy, where the plant system is represented as a network of connected hydraulic resistances (van den Honert 1948). The Hydraulic architecture (HA) has been proposed by Zimmermann (1978) as a conceptual framework to represent roots as a connected network, whose structure (connection between root segments) and function (radial and axial hydraulic resistances) define its properties. Although this concept was initially meant to describe above ground parts of the plant and in particular the sensitivity of xylem to embolism (Tyree and Ewers 1991) later the notion was more specifically developed for RS (Root hydraulic architecture or RHA) by Doussan et al. (1998a) and by Couvreur et al. (2012), amongst others (see for example the work of Cowan 1965).

Several mathematical solutions of the flow equation in a complex connected system have been developed through years. Landsberg and Fowkes (1978) developed the first analytical solution for water flow in a homogeneous single root. They also proposed a solution when regularly spaced laterals are included on a hydraulically-homogeneous root branch. However, the solution does not apply to the case of hydraulic properties varying along the root and an exact solution of the second case can be found without incorporating the laterals on the primary branch. Biondini (2008) and Roose and Schnepf (2008) also developed analytical solutions for continuous root (i.e. without segmenting the roots into small homogeneous root parts called segments) including second order roots (and higher) but they did not consider the resistance to flow along the main axis between the laterals and/or they did not include possible changes of hydraulic properties along a root branch.

Recently, Couvreur et al. (2012) developed a RS-scale model for solving water flow in RHA. In this approach, RHA is characterized with only three macroscopic (i.e. plant-scale) parameters: (1) the global hydraulic conductance of the root system ($\mathrm {K_{rs} \,[L^3 T^{-1} P^{-1}]}$), (2) the Standard Uptake Fractions ($\mathrm {SUF [-]}$), or relative distribution of water uptake under uniform soil water potential, and (3) the compensatory root water uptake conductance ($\mathrm {K_{comp} \, [L^3 T^{-1} P^{-1}]}$). The parameter $\mathrm {K_{rs}}$ defines how easily water flows through a root system. The larger the conductance, the lower the pressure drop inside the root system. The $\mathrm {SUF}$ provides weighing factors to obtain the equivalent soil water potential sensed by the plant (called $\mathrm {H_{s,eq} \, [P]}$) by averaging the distributed soil water potentials. The third plant-scale parameter $\mathrm {K_{comp}}$ controls the redistribution of root water uptake (RWU) in non-uniform soil water potential conditions (i.e. process known as compensation, or compensatory RWU, see Jarvis 2011 or Javaux et al. 2013). The advantage of this approach is that it does not rely on an explicit consideration of the RS architecture and reduces the RHA characteristric parameters to three, while keeping an exact representation of the water flow physical principles (Couvreur et al. 2012). Moreover, these macroscopic root system hydraulic parameters can be used in coupled soil–plant models (such as models of de Jong van Lier et al. 2013 or Javaux et al. 2008) to simulate the water uptake for contrasted soil and climate conditions. Leitner et al. (2014b) indeed showed that optimal root traits depend on soil and climate properties.

In a context of global climate change and expected water limitations on crop yield (Cattivelli et al. 2008), identifying key factors affecting crop water productivity is essential (Volpe et al. 2013; Passioura 2006). Root systems, with their large natural variability seem to be good candidates for crop plant improvement. Developing plant-scale indices, which quantify the ability of a RS to extract water from heterogeneous soil is a key step in the search of optimal plants under dry conditions. However, no quantitative model exists yet to link the RS hydraulic parameters to local root functional and structural properties (i.e. root angle, distance between laterals, local hydraulic resistances, etc.). Our objective is thus to provide novel mathematical models linking local root structural and functional traits to plant-scale properties of RS: $\mathrm {K_{rs}}$, $\mathrm {SUF}$ and $\mathrm {K_{comp}}$. In this study, we will focus on the first two parameters after demonstrating that $\mathrm {K_{comp}}$ is equal to $\mathrm {K_{rs}}$ under certain conditions.

To achieve this goal, the following methodology will be followed: (1) to review, adapt existing or develop new mathematical solutions of the flow equations explicitly accounting for specific local root traits in simple RS with increasing degrees of complexity; (2) to extract the upscaled hydraulic parameters from these solutions in order to (3) investigate how root traits impact them.

The structure of the paper is the following: first we develop the theory of our model with mathematical tools applied to RS with increasing complexity. We then illustrate the model functioning with two applications: the first one uses the model to derive root hydraulic parameters from experimental data; the second is theoretical and analyses the sensitivity of the macroscopic parameters of a mature maize RS to local traits. Finally, we demonstrate and discuss the application of the model in quantitative phenotyping.

## Theory

### Root water flow model

#### Water flow equation

Two local hydraulic properties characterize any root or root zone: its hydraulic radial conductivity $\mathrm {k_r \, \left[ L T^{-1}P^{-1}\right] }$ and its axial intrinsic conductance $\mathrm {k_x \, \left[ L^4 T^{-1} P^{-1}\right] }$. Here and everywhere we assume a unique value for root radial conductivity in both directions (in- and outflows).

Mass conservation for a segment of length $\mathrm {dz \, [L]}$ writes

1 $$\begin{aligned} \frac{dJ_x}{dz} = 2 \pi r q_r \end{aligned}$$

where $\mathrm {J_x \, [L^3 T^{-1}]}$ is the volumetric flow rate, $\mathrm {r \,[L]}$ is the root radius and $\mathrm {q_r \, [L T^{-1}]}$ the radial flux. We call $\mathrm {J_r \, [L^3 T^{-1}]}$ the radial volumetric flow given by:

2 $$\begin{aligned} \mathrm {J_r = 2 \pi r q_r dz} \end{aligned}$$

The axial volumetric flow rate is powered by the gradient of water xylem potential $\mathrm {H_x \, [P]}$ (defined as the sum of the gravitational and the pressure potentials):

3 $$\begin{aligned} J_x = -k_x \frac{dH_x}{dz} \end{aligned}$$

The water radial flux comes from the water potential difference between the root xylem potential and the soil–root interface $\mathrm {H_{sr} \, [P]}$:

4 $$\begin{aligned} q_r = k_r \left( H_{sr} - H_{x} \right) \end{aligned}$$

Combining Eqs. (1), (3) and (4) we obtain the water flow equation in roots:

$$\begin{aligned} \frac{d}{dz}\left( -k_x \frac{dH_x}{dz}\right) = 2 \pi r k_r \left( H_{sr} - H_{x} \right) \end{aligned}$$

This equation can be solved given that two boundary conditions are provided and the root hydraulic properties are known. The solution takes the form of a continuous function of the xylem potential which leads to the axial and radial root water flows. The flow can then be used to derive the root macroscopic parameters described in the next section.

#### Macroscopic parameters

Couvreur et al. (2012) model provides a solution based on three parameters to represent water flow in any complex RS. These three parameters are the root system conductance or $\mathrm {K_{rs} \,[L^{3} P^{-1} T^{-1}]}$, the Standard Uptake Fraction or $\mathrm {SUF \,[-]}$ and the compensatory water uptake conductance or $\mathrm {K_{comp} \, [L^3 T^{-1} P^{-1}]}$. This physically-based model of the water flow in root systems can be summarized with two main equations. The first one is the segment water uptake which is given by the sum of two processes: the water uptake in homogeneous soil conditions and the compensatory water uptake that occurs in heterogeneous conditions:

5 $$\begin{aligned} J_r = T_{act} SUF + K_{comp} \left( H_{sr}(z) - H_{s,eq} \right) SUF \end{aligned}$$

with $\mathrm {J_{r} \, [L^3 T^{-1}]}$ the radial flow, $\mathrm {T_{act} \, [L^3 T^{-1}]}$ the actual transpiration, $\mathrm {H_{sr}(z) \, [P]}$ the soil–root interface potential and $\mathrm {H_{s,eq} \, [P]}$ the soil equivalent potential felt by the plant defined as the soil–root interface potential weighted by the standard uptake fraction. The second equation describes the relation between the actual transpiration and the collar potential $\mathrm {H_{collar} \, [P]}$:

6 $$\begin{aligned} T_{act} = {K_{rs}}\left( {H_{s,eq}-H_{collar}}\right) \end{aligned}$$

What we learn from such a model is that we can simulate accurately the water uptake in any condition of any root system once we know the macroscopic parameters. As shown by the authors, these plant-scale variables can be calculated numerically. Let us first detail what they exactly mean and how we can calculate them.

The first plant-scale macroscopic parameter is the global conductance of the RS, $\mathrm {K_{rs}}$. It represents the ability of a root system to uptake water to sustain the transpiration $\mathrm {T_{act} \, [L^3 T^{-1}]}$ under a specific difference between the soil water status and the xylem plant potential and can be calculated in homogeneous soil conditions. We fix the collar potential and impose a uniform soil–root interface. In such a case, $\mathrm {H_{sr}(z) = H_{s,eq}}$ and the conductance can be obtained calculating the actual transpiration that the root system can deliver: Eq. (6) yields:

7 $$\begin{aligned} K_{rs} = \frac{T_{act}}{\left( H_{s,eq}-H_{collar}\right) } \end{aligned}$$

Note that the root system conductance can also be calculated using Thevenin theorem (1883).

The second macroscopic root system parameter is the Standard Uptake Fraction $\mathrm {SUF}$ or the normalized water uptake under uniform conditions. By definition, $\mathrm {SUF}$ is the radial flow entering each root segment divided by the collar root flow under homogeneous soil conditions. Again it can be calculated solving the water flow problem in uniform soil conditions, Eq. (5) yields:

$$\begin{aligned} SUF = \frac{J_r}{T_{act}} \end{aligned}$$

In continuous domains, we can define a new parameter characterizing the distribution of water uptake rate densities under uniform soil water potential distribution: $\mathrm {SUD \, [L^{-1}]}$) for Standard Uptake Density. $\mathrm {SUD}$ is directly related to $\mathrm {SUF}$ through the segment length $\mathrm {l_{seg} \, [L]}$:

$$\begin{aligned} SUD = \frac{SUF}{l_{seg}} \end{aligned}$$

By definition, the integration of $\mathrm {SUD}$ over the total root length yields 1. Mathematically, $\mathrm {SUD}$ is defined as:

$$\begin{aligned} SUD = \frac{2 \pi r q_r}{T_{act}} \end{aligned}$$

The third parameter is $\mathrm {K_{comp}}$, the compensatory conductance. It characterizes the ability of a plant to compensate, i.e. taking up more water in regions where it is more available (i.e. under heterogeneous soil conditions). It must be calculated under heterogeneous soil conditions. If the collar flow is null, the root water uptake/release is due to the heterogeneous soil–root potential only and controlled by $\mathrm {K_{comp}}$. In this case, Eq. (5) yields ($\mathrm {T_{act} = 0}$):

$$\begin{aligned} J_r = K_{comp} \left( H_{sr}(z) - H_{s,eq} \right) SUF \end{aligned}$$

The definition of the equivalent soil water potential slightly changes in continuous domains: $\mathrm {H_{s,eq} = \int _0^{l_{root}} H_{sr}(z) SUD(z, l_{root}) dz }$. The equivalent potential sensed by the plant is now obtained integrating the soil–root interface potential $\mathrm {H_{sr}(z) \, [P]}$ weighted by the Standard Uptake Density. The integration domain in the case of a single root is between 0 and the root length $\mathrm {l_{root} \, [L]}$ as shown here or in case of complex root systems it is performed on the whole root system length.

### Single homogeneous root

**Fig. 1:**
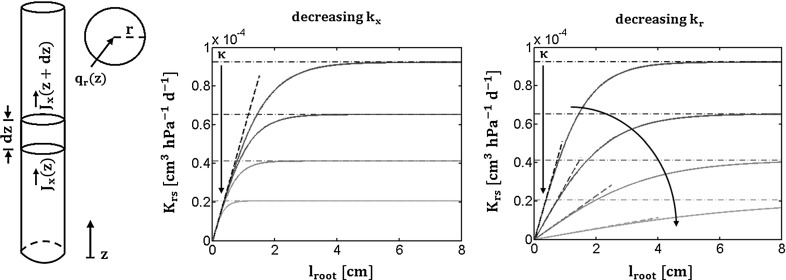
Continuous model for a homogeneous root: Layout of the uniform root simulated (*left*). The second (*center*) and third (*right*) panels illustrate the sensitivity of the $\mathrm {K_{rs}}$ macroscopic parameter decreasing the axial intrinsic conductance and radial conductivity, respectively. For details, see text

Let us consider a simple RS made of a single root branch with uniform hydraulic properties. The RS has a radius $\mathrm {r}$ and a length $\mathrm {l_{root} \, [L]}$ and is oriented along the z-axis (a list of the principal symbols can be found in “Appendix 1”). $\mathrm {z}$ is defined as the position along the root, zero at the root tip, positive towards the plant collar by convention. Let us assume that the water potential at the soil–root interface $\mathrm {H_{sr}(z) \, [P]}$ is uniform and equal to $\mathrm {H_{soil} \, [P]}$. The xylem water potential in the root collar is called $\mathrm {H_{collar} \, [P]}$. This situation is shown in Fig. 1 (left).

The analytical solution for water flow in a uniform root was proposed by Landserg and Fowkes (1978) when a constant collar potential and no-flux at the tip are imposed:

$$\begin{aligned} H_x (z, l_{root})= & {} H_{soil}+ \left( {H_{collar}-H_{soil}}\right) \frac{cosh\left( \tau z\right) }{cosh\left( \tau l_{root}\right) }\\ J_x (z, l_{root})= & {} k_x \tau \left( {H_{soil}-H_{collar}}\right) \frac{sinh\left( \tau z\right) }{cosh\left( \tau l_{root}\right) }\\ J_r (z, l_{root})= & {} dz k_x \tau ^2 \left( {H_{soil}-H_{collar}}\right) \frac{cosh\left( \tau z\right) }{cosh\left( \tau l_{root}\right) } \end{aligned}$$

where cosh, sinh and tanh are the hyperbolic cosine, sine and tangent functions, respectively. We call $\mathrm {\tau = \sqrt{\frac{2 \pi r k_r}{k_x}} \, [L^{-1}]}$. This parameter is similar to $\alpha$ introduced by Alm et al. (1992) and Landsberg and Fowkes (1978). See “Appendix 2” for mathematical details (adapted from Landsberg and Fowkes 1978).

Since the equivalent potential in case of uniform soil water potential yields:

$$\begin{aligned} H_{s,eq} = H_{soil} \end{aligned}$$

we obtain for the root conductance $\mathrm {K_{rs}(l_{root})}$ using its definition (7) ($\mathrm {T_{act}}$ is the axial flow at the root collar, i.e. in $\mathrm {z = l_{root}}$):

8 $$\begin{aligned} K_{rs}(l_{root}) \overset{\varDelta }{=} \frac{J_x(z = l_{root},l_{root})}{H_{soil}-H_{collar}} = k_x \tau \frac{sinh\left( \tau l_{root}\right) }{cosh\left( \tau l_{root}\right) } = \kappa tanh(\tau l_{root}) \end{aligned}$$

with

9 $$\begin{aligned} \kappa = \tau k_x = \sqrt{2 \pi r k_r k_x} \, \mathrm {[L^{3} P^{-1} T^{-1}]} \end{aligned}$$

Interestingly in case of non-limiting xylem conductance and/or small roots:

$$\begin{aligned} {\tau l_{root}<< 1 \iff tanh(\tau l_{root}) \simeq \tau l_{root}} \end{aligned}$$

the global conductance can be approximated by a linear relation:

10 $$\begin{aligned} K_{rs}(l_{root}) \simeq 2\pi r k_r l_{root} \end{aligned}$$

In this case the global conductance of the root is proportional to the total root surface: $\mathrm {2\pi r l_{root}}$. In case of very long roots and/or xylem conductance limiting conditions ($\mathrm {\tau l_{root}>> 1}$), an asymptotic value $\mathrm {\kappa }$ is reached:

11 $$\begin{aligned} K_{rs}(l_{root}) \rightarrow \kappa \end{aligned}$$

This asymptotic value of conductance implies that for given local hydraulic properties and water potential difference between the soil and the root collar, there is a maximal flow rate. This suggests that there is an optimal length beyond which the root conductance does not increase significantly. At that point plant marginal interest in investing structural carbon in the root for water acquisition is not significant.

Centre and right panels of Fig. 1 illustrate the sensitivity of the global conductance of the uniform root to changes in axial and radial conductivities. In these subplots Eqs. (8), (10) and (11) are plotted as blue solid lines, dark/grey linear relations and dark/grey dashed-dotted horizontal lines, respectively. The dark arrows indicate the decreasing trend of $\mathrm {K_{rs}}$ curves when decreasing root hydraulic conductivities (resp. axial and radial conductivities in central and right subplots).

Note that for this figure and all other figures in this study we used maize root conductivities values obtained by Doussan et al. (1998b) and architectural traits (radius, internodal distances) from RootTyp (Pags et al. 2004) parametrized for maize (Couvreur et al. 2012). In particular young segments have been considered for the first figures.

**Fig. 2:**
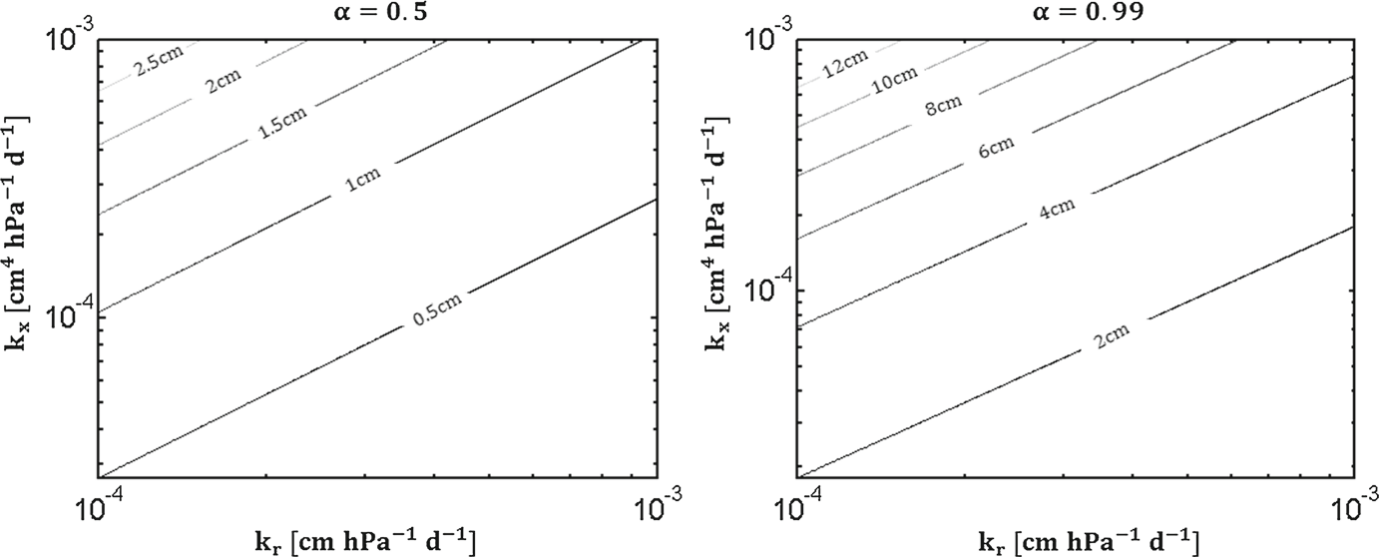
l$^*(\alpha )$: Root length necessary to reach two different $\mathrm {\mathbf {\alpha }}$ fractions of $\mathrm {\kappa }$ as a function of the root local conductivities

Since a maximal flow rate exists for a given water potential difference between the soil and the collar we can define the fraction $\mathrm {\alpha \, [-]}$ of the actual flow to the maximal one. By definition it is the root conductance divided by its asymptotic value. Inversely, we derive the root length $\mathrm {l^*}$ needed to reach a specific ratio of the maximal flow.

$$\begin{aligned} \alpha (l^*) \overset{\varDelta }{=} \frac{\kappa tanh(\tau l^*)}{\kappa } = tanh(\tau l^*) \iff l^*(\alpha ) = \frac{atanh(\alpha )}{\tau } \end{aligned}$$

with atanh the inverse hyperbolic tangent function. This solution allows us to calculate the length necessary to reach a specific conductance such as $\mathrm {\alpha = 0.5}$, or $\mathrm {\alpha = 0.99}$. This length depends on the root radial and axial conductivity, as illustrated in Fig. 2. This equation puts the bases of allometric relations for roots allowing to relate optimal root branch length to their local properties.

The second root system parameter is the Standard Uptake Density or $\mathrm {SUD}$. Using its definition we obtain:

12 $$\begin{aligned} SUD (z, l_{root}) \overset{\varDelta }{=} \frac{2 \pi r q_r(z, l_{root})}{K_{rs} \left( H_{soil}-H_{collar}\right) } = \tau \frac{cosh\left( \tau z\right) }{sinh(\tau l_{root})} \end{aligned}$$

We can also approximate the Standard Uptake Density by a Taylor series ($\mathrm {\tau l_{root}<< 1 \Rightarrow \tau z<< 1}$, $\mathrm {\forall z}$):

13 $$\begin{aligned} SUD(z, l_{root}) \simeq \frac{1 }{l_{root}} \left( 1 + \frac{\left( \tau z \right) ^2}{2} \right) \end{aligned}$$

In this case the Taylor approximation of the the distribution of water uptake rate densities is a parabola meaning that the maximal uptake occurs at the proximal part of the uniform root ($\mathrm {z = l_{root}}$).

Under non-limiting xylem conductance conditions though, the $\mathrm {SUD}$ is almost uniform along the root and equals to $\mathrm {\frac{1}{l_{root}}}$. We obtain the latter result by neglecting the quadratic dependence of $\mathrm {SUD}$ in z:

$$\begin{aligned} SUD(z, l_{root}) \simeq \frac{1 }{ l_{root}} \left( 1 + \frac{\left( \tau z \right) ^2}{2} \right) \simeq \frac{1}{l_{root}} \end{aligned}$$

**Fig. 3:**
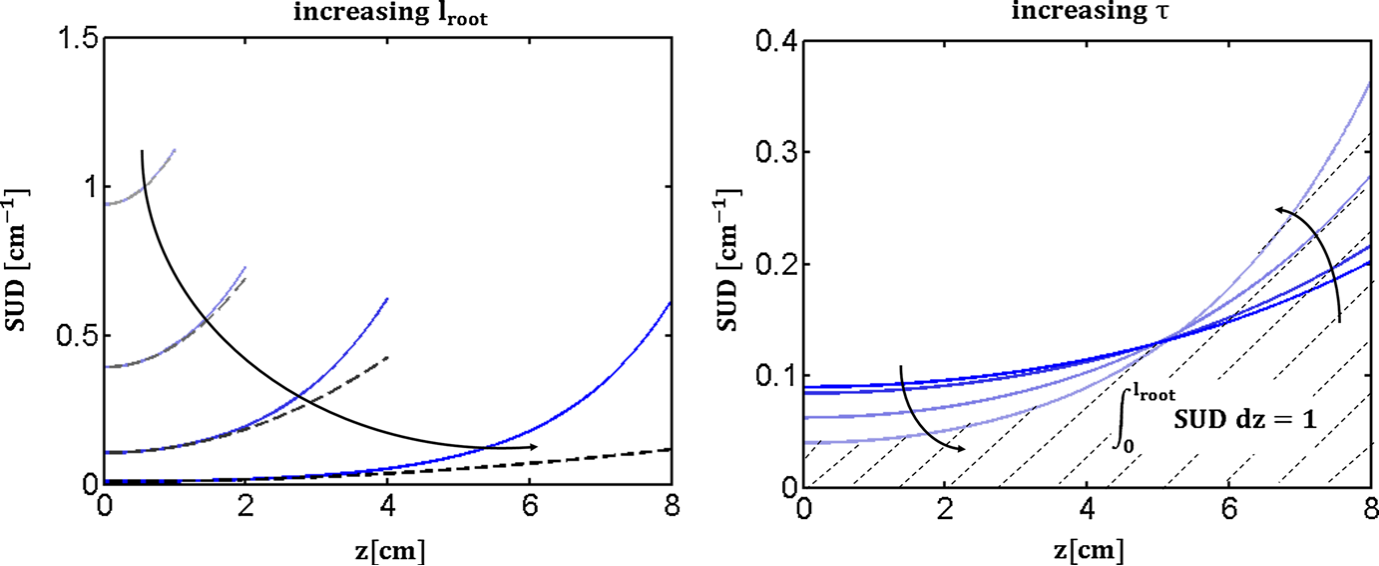
Continuous model for a homogeneous root: sensitivity analysis of the $\mathrm {SUD}$ macroscopic parameter according to changes in $\mathrm {l_{root}}$ and $\mathrm {\tau }$ parameters. For details, see text

Figure 3 illustrates the uptake rate relative density in standard conditions and its sensitivity to $\mathrm {l_{root}}$ and $\mathrm {\tau }$, respectively. In the left panel, the lighter blues solid line are smaller roots. The gray dashed lines are the harmonic approximations predicted from Eq. (13). The smaller the $\tau l$ product the better the approximation. In the right subplot $\mathrm {\tau }$ intrinsic property of the root is gradually increased. The smaller the radial to axial conductivity ratio the more homogeneous the water uptake. Indeed the axial resistance is less and less limiting so that the radial barrier becomes progressively the highest resistance to root water flow. The area below the curve always remains equal to 1.

To derive the third macroscopic parameter ($\mathrm {K_{comp}}$), new boundary conditions have to be imposed. Indeed this parameter only plays a role in non-uniform soil water potential conditions. We impose now non-uniform soil–root interface potential, no flux at the tip and at the collar, which write:

$$\begin{aligned} \left\{ \begin{array}{l} H_{sr}(z) = \alpha exp \left( \beta z \right) \\ J_{x}(z = 0,l_{root}) = 0 \\ J_x(z = l_{root},l_{root})= 0 \\ \end{array} \right. \end{aligned}$$

In this case the solution for the xylem water potential distribution becomes:

$$\begin{aligned} H_x (z, l_{root}) = \frac{\tau ^2}{\tau ^2 - \beta ^2} \alpha exp \left( \beta z \right) + c_1 exp\left( \tau z\right) +c_2 exp\left( -\tau z\right) \end{aligned}$$

with $\mathrm {c_1}$ and $\mathrm {c_2}$ that can be obtained using previously described root boundary conditions:

$$\begin{aligned} \left[ \begin{array}{c} c_1 \\ c_2 \\ \end{array} \right] = \left[ \begin{array}{c c} \tau &{} -\tau \\ \tau exp(\tau l_{root}) &{} -\tau exp(-\tau l_{root}) \end{array} \right] ^{-1} \left[ \begin{array}{c} \frac{-\alpha \beta \tau ^2}{\tau ^2 - \beta ^2} \\ - \frac{-\alpha \beta \tau ^2}{\tau ^2 - \beta ^2} exp(\beta l_{root})\\ \end{array} \right] \end{aligned}$$

Interestingly, we find:

$$\begin{aligned} \frac{J_r}{SUF} \overset{\tau l_{root} \rightarrow 0}{\simeq } 2 \pi r k_r l_{root} \left( H_{sr} - H_{s,eq} + c.\mathcal {O} \left( \tau l_{root} \right) \right) \end{aligned}$$

with $\mathrm {c}$ a constant depending on the root properties and soil conditions. So that $\mathrm {K_{comp}}$ is equal to $\mathrm {K_{rs}}$ (see Eq. 10) as long as the xylem conductance is non-limiting, as shown numerically by Couvreur et al. (2012). When xylem conductance is limiting, $\mathrm {K_{comp}}$ cannot be defined analytically in a simple way, but in tested maize and wheat HA a value close to $\mathrm {K_{rs}}$ predicts compensatory uptake satisfyingly well (Couvreur et al. 2014). In the following sections we only consider $\mathrm {K_{rs} = K_{comp}}$ for sake of simplicity but we always can use the previous equation to evaluate this approximation’s accuracy.

To summarize this first theoretical section, from the physical equations of water flow in a root cylinder with uniform hydraulic properties (solutions of Landsberg and Fowkes 1978), we derived analytical expressions for the macroscopic root hydraulic parameters of Couvreur et al. (2012) model in terms of local hydraulic properties. These plant-scale parameters depend on both hydraulic (axial and radial conductivities) and geometrical traits (length, radius). Particularly, two local hydraulic properties determine the macroscopic parameters: $\mathrm {\kappa }$ provides the asymptotic root conductance, and $\mathrm {\tau }$ determines how quickly a growing root approaches its asymptotic conductance, and how uniform is the water uptake profile along the root. These local properties do not depend on the boundary conditions. They are intrinsic root properties, just like the macroscopic parameters.

### Root with heterogeneous properties

We consider now a root cylinder made of several homogeneous root zones. Each zone is characterized by its own hydraulic radial conductivity $\mathrm {k_{r,i}}$ and axial conductance $\mathrm {k_{x,i}}$ with i varying from one to N the total zone number. To calculate the macroscopic parameters of the entire root we first focus on one of the root zones called generically hereafter i. For all zones except the apical one, the upstream root part can be seen as an initial conductance attached to the considered zone. The root zone under focus has a root length $\mathrm {l_{root,i}}$ and the position $\mathrm {z_i \, [L]}$ along the root portion varies between 0 and $\mathrm {l_{root,i}}$. We define the upstream or distal end of the root zone as the position closest to the root tip ($\mathrm {z = 0}$) and the downstream or proximal end as the end of the root zone ($\mathrm {z = l_{root,i}}$).

We can define an asymptotic conductance $\mathrm {\kappa _{i}}$ and a convergence speed $\mathrm {\tau _{i}}$ for each root zone. Therefore we apply the same methodology than in the previous sections except for the boundary conditions that are different than no flux at the lower end of the root cylinder to solve the water flow equation. For the ith zone, the boundary conditions write:

$$\begin{aligned} \left\{ \begin{array}{l} H_x(z_i = l_{root,i},l_{root,i})= H_{x,i} \\ J_x(z_i = 0,l_{root,i}) = J_{i-1} \\ \end{array} \right. \end{aligned}$$

where the first variable under parentheses represents the location within the ith zone, and the second one, the length of this zone). $\mathrm {H_{x,i} \, [P]}$ is the proximal pressure head and $\mathrm {J_{i-1} \, [L^3 T^{-1}]}$ is the distal flow. The latter condition is used to simulate the presence of an upstream root cylinder below the current one (distal end). Using the methodology developed in the “Appendix 2” to solve the flow equation, we obtain after simplification:

14 $$\begin{aligned} H_x(z_i, l_{root,i}) = H_{soil} + \left( H_{x,i} - H_{soil} \right) \frac{cosh(\tau _i z)}{cosh(\tau _i l_{root,i})} + \frac{J_{i-1}}{\kappa _i} \frac{sinh(\tau _i l_{root,i} - \tau _i z)}{cosh(\tau _i l_{root,i})} \end{aligned}$$

and

$$\begin{aligned} J_x(z_i, l_{root,i}) = -k_{x,i} \frac{d H_x}{d z_i} = \varDelta H_i \kappa _i \frac{sinh(\tau _i z_i)}{cosh(\tau _i l_{root,i})} + J_{i-1} \frac{cosh(\tau _i l_{root,i} - \tau _i z_i)}{cosh(\tau _i l_{root,i})} \end{aligned}$$

with $\mathrm {\varDelta H_i = H_{soil} - H_{x,i} \, [P]}$. It can be seen that these two equations verify the boundary conditions.

Using the macroscopic parameters definitions given in a previous section (see 2.1.2) we obtain for the root conductance $\mathrm {K_{rs}(l_{root,i})}$ defined as the conductance of the entire root system including the distal part and the root segment with length $\mathrm {l_{root,i}}$:

15 $$\begin{aligned} K_{rs,i}(l_{root,i})\overset{\varDelta }{=} \frac{J_x(l_{root,i}, l_{root,i})}{\varDelta H_i } = \kappa _i tanh(\tau _i l_{root,i}) + \frac{J_i}{\varDelta H cosh(\tau _i l_{root,i})} \end{aligned}$$

The conductance of the distal root zone in $\mathrm {z_i = 0}$ is given by:

$$\begin{aligned} K_{rs,i-1} \overset{\varDelta }{=} \frac{J_i}{H_{soil} - H_x (z_i = 0,l_{root,i})} \end{aligned}$$

The conductance of the zone i–1 is indeed by definition the flow $\mathrm {J_i}$ which arrives to segment i divided by the potential difference between the soil and the xylem at the connection between the root cylinders, $\mathrm {z_i = 0}$ in this case. When $\mathrm {K_{rs,i-1}}$ with $\mathrm {H_x(z_i, l_{root,i})}$ estimated by Eq. (14) is inserted in Eq. (15), it yields:

16 $$\begin{aligned} K_{rs,i}(l_{root,i})= & {} \kappa _i \left[ tanh(\tau _i l_{root,i}) \right. \nonumber \\&\left. + \frac{K_{rs,i-1}}{cosh(\tau _i l_{root,i})\left( K_{rs,i-1} sinh(\tau _i l_{root,i}) + {\kappa _i} cosh(\tau _i l_{root,i})\right) } \right] \nonumber \\ \end{aligned}$$

Let us point that, if the zone length we are focusing on is zero, the root conductance is simply $\mathrm {K_{rs,i-1}}$, the conductance of the upstream zone.

Similarly a new solution is obtained for the $\mathrm {SUD}$. The standard uptake density along the considered root fraction is independent of the soil water potential:

17 $$\begin{aligned} SUD (z_i , l_{root,i}) = \tau _i \left[ \frac{\kappa _i cosh(\tau _i z_i) + K_{rs,i-1}sinh(\tau _i z_i)}{\kappa _i sinh(\tau _i l_{root,i}) + K_{rs,i-1} cosh(\tau _i l_{root,i})} \right] \end{aligned}$$

These solutions (Eqs. 16, 17) are a generalisation of the solutions of the homogeneous root Eqs. (8) and (12) to roots with several differentiated zones (straightforward with $\mathrm {K_{rs,0} = 0}$).

To summarize, we just have presented a method that can be used to assemble the elementary building blocks (i.e. the zones) into complex hydraulic systems. We can now generalize for a compartmented root of total length $\mathrm {l_{root}}$ made of N differentiated zones. The locations of the connections between zones are called $\mathrm {l_i}$, which corresponds to the cumulative sums of the different zone lengths $\mathrm {l_{root,i}}$. When continuity of both axial fluxes and potentials is imposed at the junction between distinct zones as ’intermediary’ conditions and when no flux at the root tip, i.e. $\mathrm {J_x(z = 0,l_{root})= 0}$ and a potential top boundary condition $\mathrm {H_x(z=l_{root},l_{root}) = H_{collar}}$ are imposed just as in the first section, a unique solution can be found. Mathematically we impose from the tip to the collar:

18 $$\begin{aligned} \left\{ \begin{array}{l} J_x(z = 0, l_{root}) = 0 \\ H_x (z = l_i, l_{root})|_i = H_x (z = l_{i}, l_{root})|_{i+1} \qquad \qquad \forall i \in \left[ 1,N-1\right] \\ J_x(z = l_i, l_{root})|_i = J_x(z = l_{i}, l_{root})|_{i+1} \qquad \qquad \forall i \in \left[ 1,N-1\right] \\ H_x (z = l_{root}, l_{root}) = H_{collar} \\ \end{array} \right. \end{aligned}$$

where $\mathrm { \dots |_i}$ means that the function is evaluated in the ith zone.

The solution of the flow equation inside each root zone is given by the general solution (19) applied to the boundary conditions (18).

19 $$\begin{aligned}&H_x(z,l_{root}) \nonumber \\&= \left\{ \begin{array}{l} H_{soil} + c_1 exp\left( \tau _1 z\right) +c_2 exp\left( -\tau _1 z\right) , z \in \left[ 0, l_1 \right] \\ H_{soil} + c_3 exp\left( \tau _2 (z - l_1)\right) +c_4 exp\left( -\tau _2 (z-l_{1}) \right) , z \in \left[ l_1, l_2\right] \\ \vdots \\ H_{soil} + c_{2N-1} exp\left( \tau _N (z-l_{N-1})\right) +c_{2N} exp\left( -\tau _N (z-l_{N-1}) \right) , z\in \left[ l_{N-1}, l_{N}\right] \end{array} \right. \end{aligned}$$

where $\mathrm {\mathbf {c}}$ is the vector of coefficients that can be determined by the boundary conditions (18). For instance, if we consider two zones equations (18) become:

$$\begin{aligned} \left\{ \begin{array}{l} J_x(z = 0, l_{root}) = 0 \\ H_x (z = l_1, l_{root})|_1 = H_x (z = l_{1}, l_{root})|_2 \\ J_x(z = l_1, l_{root})|_1 = J_x(z = l_{1}, l_{root})|_2 \\ H_x (z = l_{root}, l_{root}) = H_{collar} \\ \end{array} \right. \end{aligned}$$

Or

$$\begin{aligned} \left\{ \begin{array}{l} -k_{x1} \left( c_1 \tau _1 -c_2 \tau _1 \right) = 0\\ c_1 exp(\tau _1 l_1) + c_2 exp( - \tau _1 l_1) = c_3 + c_4\\ -k_{x1} \tau _1 \left( c_1 exp(\tau _1 l_1) - c_2 exp(-\tau _1 l_1) \right) = -k_{x2} \tau _2\left( c_3 - c_4 \right) \\ H_{soil} + c_3 exp(\tau _2 (l_2-l_1) + c_4 exp(-\tau _2 (l_2 - l_1) = H_{collar} \\ \end{array} \right. \end{aligned}$$

This set of four boundary conditions can be rewritten in matrix notations factorizing the coefficients $\mathrm {c_i}$:

$$\begin{aligned} \mathbf {R}*\mathbf {c} = \mathbf {H} \end{aligned}$$

with:

$$\begin{aligned} \mathbf {c}= & {} \left[ \begin{array}{l} c_1 \\ c_2 \\ c_3 \\ c_4 \\ \end{array} \right] \\ \mathbf {R}= & {} \left[ \begin{array}{c c c c} \tau _1 &{} -\tau _1 &{} 0 &{} 0 \\ exp\left( \tau _1 l_1 \right) &{} exp\left( -\tau _1 l_1 \right) &{} -1 &{} -1 \\ \kappa _1 exp\left( \tau _1 l_1 \right) &{} -\kappa _1 exp\left( -\tau _1 l_1 \right) &{} -\kappa _2 &{} \kappa _2 \\ 0 &{} 0 &{} exp\left( \tau _2 (l_2-l_1) \right) &{} exp\left( -\tau _2 (l_2-l_1) \right) \\ \end{array} \right] \\ \mathbf {H}= & {} \left[ \begin{array}{c} 0 \\ 0 \\ 0 \\ H_{collar} - H_{soil} \\ \end{array}\right] \end{aligned}$$

This mathematical development allows the splitting of any root in an arbitrary number of differentiated homogeneous zones by calculating the vector $\mathrm {\mathbf {c}}$ using:

$$\begin{aligned} \mathbf {c} = \mathbf {R^{-1}} \mathbf {H} \end{aligned}$$

It allows solving the water flow equation in a single root branch with hetereogeneous properties. By inserting the values of vector $\mathrm {\mathbf {c}}$ in Eq. (19), we obtain the xylem potential everywhere inside the root branch and consequently the axial and radial fluxes by applying Eqs. (29) and (30), respectively. The macroscopic parameters are then obtained using their definition (8) and (12).

**Fig. 4:**
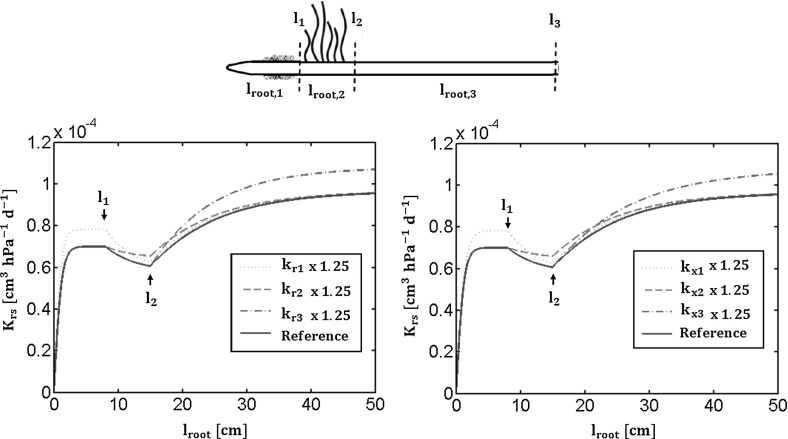
Root with heterogeneous properties: the top-drawn root is a layout of a three-compartmented root. The two subplots represent the root global conductance as a function of different local properties (*dark blue solid line*) and its sensitivity to the local radial (*lighter blue lines*, left subplot) or axial (*lighter blue lines*, right subplot) conductivities

Figure 4(top) shows a RS made of three zones, each of them having homogeneous hydraulic properties. In the left subplot the root global conductance is shown as a function of the total root length (dark blue solid line). The $\mathrm {K_{rs}}$ can be calculated at each location z between the tip and $\mathrm {l_{root}}$, and will depend on the hydraulic properties of the root segment and zones between 0 and the location z. How the $\mathrm {K_{rs}}$ will change with z will depend on the values of the root properties along the root branch. If we change each of the radial or axial hydraulic conductivities separately, $\mathrm {K_{rs}}$ will change accordingly in a different way, as showed in Fig. 4. The sensitivity of this macroscopic parameter to the different radial conductivities when increasing the local hydraulic traits by 25% are plotted (light blue solid lines). In the right subplot we present the same sensitivity analysis of this root conductance to each local hydraulic axial conductances. The reference values are the different hydraulic conductivity plateau obtained by Doussan et al. (1998b) for a maize lateral root.

The sensitivity of this macroscopic parameter to the different radial conductivities when increasing the local hydraulic traits by 25% are also plotted (light blue solid lines). In the right subplot we present the same sensitivity analysis of this root conductance to each local hydraulic axial conductances. Increasing by 25% the radial or axial conductivity of one of the zones affects in different ways the global conductance according to the root length.

**Fig. 5:**
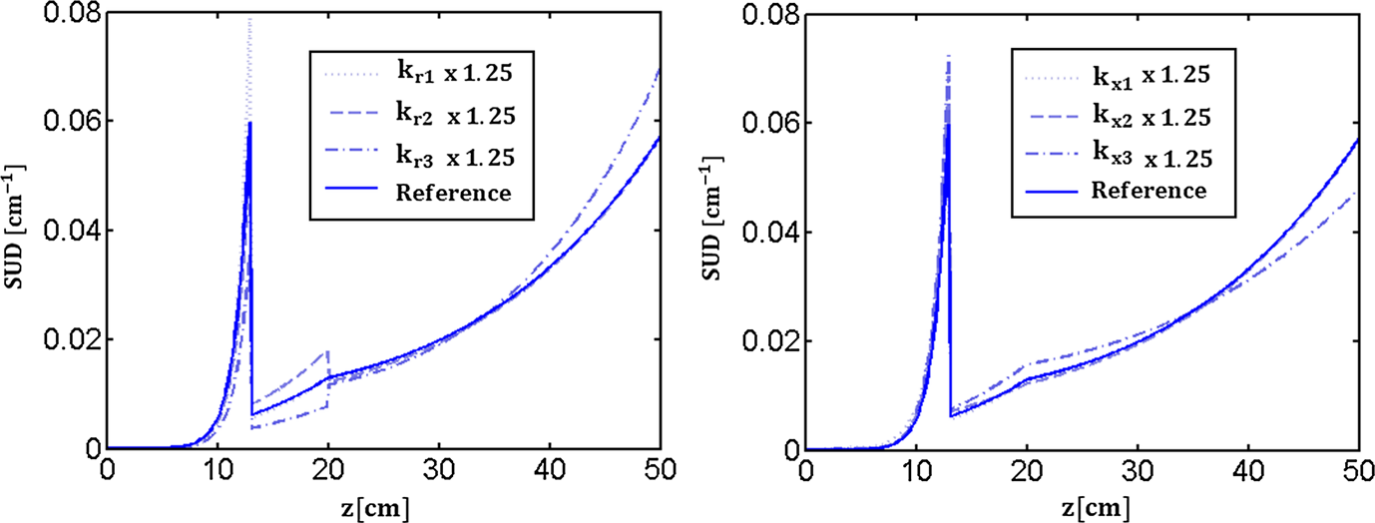
Root with heterogeneous properties: SUD along a three-zones root (*dark solid line*) and sensitivity of the normalized uptake to the local radial (left subplot) and axial (right subplot) hydraulic conductivities (*lighter blue lines*)

Figure 5 shows SUD in the case of heterogeneous root properties and its sensitivity. Here again we consider a three-zoned root. In subplots b and c, the uptake fraction density is plotted as a function of the position along the root (blue solid line). We clearly see the locations of the transition between zones. By definition the integral remains equal to unity. The sensitivity of the normalized uptake is plotted when local radial conductivity of each zone is respectively increased by 25% as compared to the generic hydraulic properties obtained by Doussan et al. (1998b) (light blue dashed, dotted and dash-dotted lines, from tip to collar zones). In the right subplot the impact of an increase of the axial conductances of each zone is shown (light blue dashed, dotted and dash-dotted lines from tip to collar zones).

### Root system with laterals

Unlike the uniform root or the root with homogeneous zones, no solution of the water flow equation could be found for a root bearing regularly spaced laterals. Landsberg and Fowkes (1978) suggested an approximation when incorporating these laterals all over the principal root length by increasing the radial conductivity of the principal root. Yet it is possible to find the conductance of this idealized root system using a discrete approach. The mathematical solution of this problem is not straightforward and we will introduce it progressively. In a first step, we present such a discrete model, its parameters and equations (Sect. 2.4.1). We then apply and solve it for the simplest case: the uniform root (Sect. 2.4.2). We show that the discrete root model converges to the exact continuous model for sufficiently small root segments. Finally we repeat the methodology for the more complex case of a root bearing laterals (Sect. 2.4.3).

#### Discrete model

In the discrete approach, we consider that the basic element is a 1D root segment of length $\mathrm {l_{seg} \, [L]}$ in which local hydraulic properties are considered as uniform. Each single segment (numbered n) has a radial ($\mathrm {K_{r,n} \, [L^{3} P^{-1} T^{-1}]}$) and an axial ($\mathrm {K_{x,n} \, [L^{3} P^{-1} T^{-1}]}$) hydraulic conductance that depends on the hydraulic and geometric root segment properties as:

$$\begin{aligned} \left\{ \begin{array}{l} K_r=2 \pi r l_{seg} k_r \\ K_x=\frac{k_x}{l_{seg}} \\ \end{array} \right. \end{aligned}$$

The segment radial conductance is thus defined as the radial conductivity multiplied by the segment surface while the root segment axial conductance is obtained by dividing the intrinsic axial conductance by the segment length. Note that we now use the subscript n instead of i to clearly distinguish root zones (that exhibit contrasted root hydraulic properties, see previous section) and root segments (that may have identical root hydraulic properties, as it will be shortly developed). Using the segment conductances, the flow in the segmented root systems is approximated for the segment numbered n (supposed here not linked to lateral roots) by:

$$\begin{aligned} \left\{ \begin{array}{l} J_{r,n} = K_{r,n} \left( H_{sr,n} - H_{x,n} \right) \\ J_{x,n} = K_{x,n} \left( H_{x,n-1} - H_{x,n} \right) \\ \end{array} \right. \end{aligned}$$

with $\mathrm {H_{sr,n}}$ the water potential at the soil root interface, $\mathrm {H_{x,n}}$ and $\mathrm {H_{x,n-1}}$ the water xylem potential inside the segment n and n–1 respectively. $\mathrm {J_{r,n}}$ and $\mathrm {J_{x,n}}$ are the radial and axial flows in the nth segment. The potential as well as the radial and axial flow are considered as uniform inside the root segment of finite length.

**Fig. 6:**
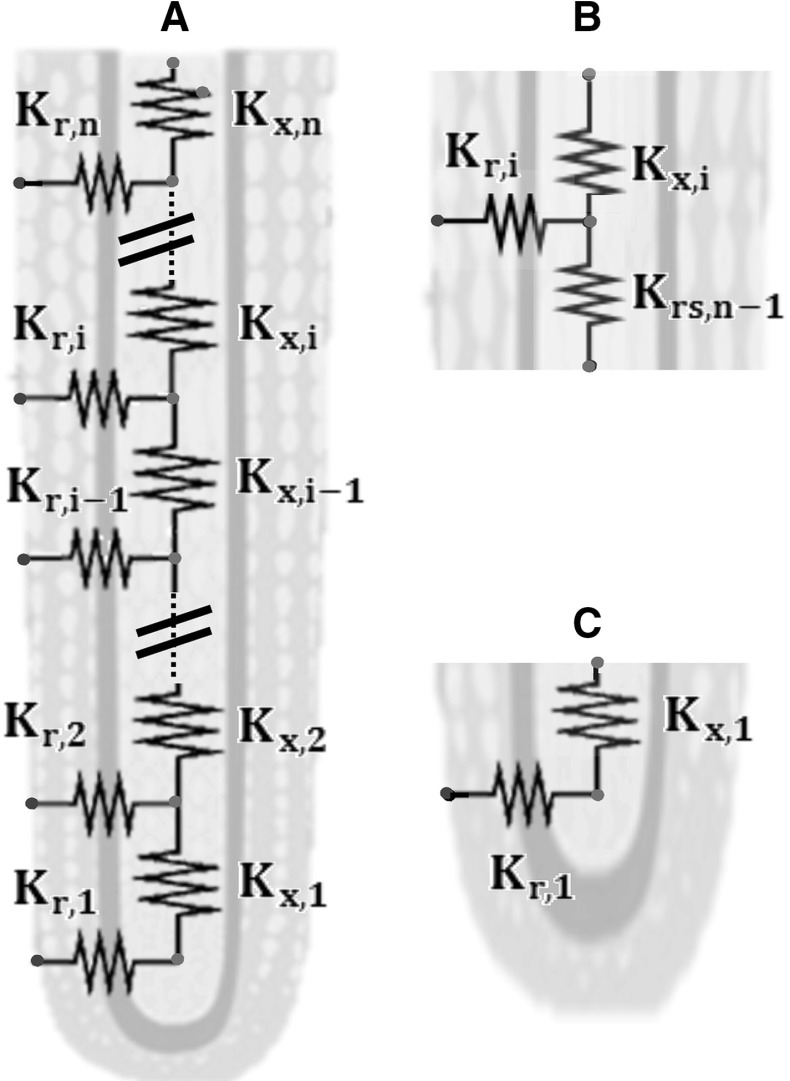
Conceptual models used for the discrete model of the homogeneous root: overview of the simple homogeneous root made of n segments (**a**) with details on the recurrence relationship (**b**) and the first recurrence (**c**)

#### Discretized uniform root branch

A root branch can be conceptualized as a recurrence of the diagram represented in Fig. 6b whose initial value is illustrated in Fig. 6c. Mathematically, the equivalent conductance for $\mathrm {n}$ segments $\mathrm {K_{rs,n}}$ is:

$$\begin{aligned}K_{rs,n}= \left\{ \begin{array}{l} \left( \frac{1}{K_{r,n}}+\frac{1}{K_{x,n}} \right) ^{-1}, \text { n} = 1 \\ \left( {\frac{1}{K_{x,n}}+\frac{1}{K_{r,n} + K_{rs,n-1}}}\right) ^{-1}, \, \mathrm{elsewhere} \\ \end{array} \right. \end{aligned}$$

The derivation of the discrete problem from continuous approach is given in the “Appendix 3”.

Note that in the specific case of homogeneous root: $\mathrm {K_{r,n}=K_r, \forall n}$ (and the same for all $\mathrm {K_{x,n}}$. It can be demonstrated that the root conductance for $\mathrm {n}$ segments is given by:

20 $$\begin{aligned} K_{rs,n}= \frac{\left[ \left( \frac{\kappa _+}{\kappa _-}\right) ^{2}\right] ^{n} - 1 }{ \frac{1}{\kappa _-}\left[ \left( \frac{\kappa _+}{\kappa _-}\right) ^{2}\right] ^{n} - \frac{1}{\kappa _+}} \begin{array}{l} \overset{n \rightarrow \infty }{\xrightarrow {}} \kappa _+ \overset{K_x>> K_r}{\xrightarrow {}} \sqrt{K_r K_x} = \sqrt{2 \pi r k_r k_x} \end{array} \end{aligned}$$

where $\mathrm {\kappa _{-} \, [L^ {3} P^{-1} T^{-1}]}$ and $\mathrm {\kappa _{+} \, [L^{3} P^{-1} T^{-1}]}$ are function of the radial and axial conductances:

21 $$\begin{aligned} \left\{ \begin{array} {l} \kappa _- = \frac{-K_r-\sqrt{K^2_r+4K_rK_x}}{2} <0 \\ \kappa _+=\frac{-K_r+\sqrt{K^2_r+4K_rK_x}}{2} >0 \\ \end{array} \right. \end{aligned}$$

Demonstration is given in “Appendix 4”. $\mathrm {\kappa _+}$ is the asymptotic value of conductance: for a homogeneous root, Eq. (20) shows that the apparent conductance tends to $\mathrm {\kappa _+}$, when the number of segments (or the root branch length) tends to infinity, as previously shown in Fig. 1 and Eq. (9). For the first limit of Eq. (20) to be verified, $\mathrm {\kappa _+}$ must have a smaller absolute value than $\mathrm {\kappa _-}$. But this is always the case since, in absolute values, according to their definitions, the former is the difference of two positive terms and the latter the sum of the same terms. Note that for $\mathrm {l_{seg} \rightarrow 0}$, $\mathrm {\kappa _+}$ goes to the exact asymptotic conductance given in Eq. (9) which is the second limit of Eq. (20).

If we define

22 $$\begin{aligned} \mathrm {\chi } = \left( \frac{\kappa _+}{\kappa _-}\right) ^{2} \end{aligned}$$

Eq. (20) simply becomes:

$$\begin{aligned} K_{rs,n}= \frac{\chi ^{n} - 1 }{ \frac{1}{\kappa _-}\chi ^{n} - \frac{1}{\kappa _+}}. \end{aligned}$$

$\mathrm {\chi }$ determines the rate of convergence of the root conductance towards its asymptotic value $\mathrm {\kappa _+}$.

It is worth mentioning that for $\mathrm {l_{seg}} \rightarrow 0$, $\mathrm {\kappa _{+}} \rightarrow \kappa$ and $\mathrm {K_{rs,n}} \rightarrow K_{rs} (nl_{seg})$. This implies that the size of the segments that lead to a certain accuracy of the discrete solution can be derived.

A finite difference solution of $\mathrm {SUF}$ for a homogeneous root can be found in function of the $\mathrm {\frac{k_r}{k_x}}$ ratio (and on the considered segment length $\mathrm {l_{seg}}$). It leads to an equation equivalent to the one found in the continuous solution section when the segment length is small enough. Details of the mathematical development are given in “Appendix 5”.

#### Discretized root branch with laterals

For RS with lateral roots, no continuous solution of $\mathrm {H_x}$ and $\mathrm {J_r}$ was found but a recurrence series can still be used to obtain the macroscopic hydraulic parameters. In this series radial conductivity and axial conductance of the principal root are assumed to be uniform as well as the conductance of lateral roots. Lateral roots can be reduced to their effective total conductance $\mathrm {K_{lat} \, [L^3 T^{-1} P^{-1}]}$, each connected in parallel to the principal root. Note that the lateral conductance $\mathrm {K_{lat}}$ is assumed to be constant. This assumption makes sense because it was demonstrated in the two previous sections that beyond a certain length the conductance of a uniform or heterogeneous root branch does not change dramatically. We call $\mathrm {d_{inter} \, [L]}$ the distance between two successive laterals. The link with the number of internodal segments ($\mathrm {N_{inter}}$) is:

$$\begin{aligned} d_{inter} = N_{inter} l_{seg} \end{aligned}$$

**Fig. 7:**
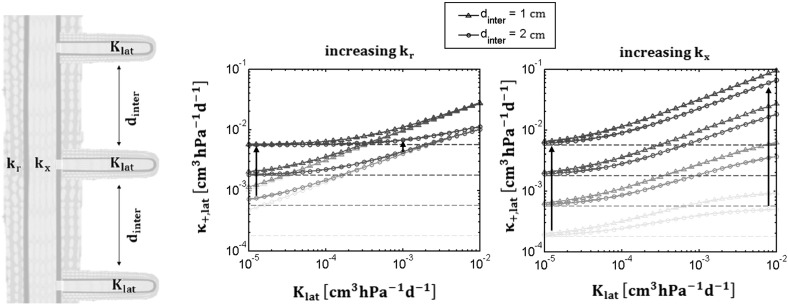
Root system with laterals: the *left panel* illustrate the conceptual root system. The two other subplots show $\mathrm {\kappa _{+,lat}}$ as a function of $\mathrm {K_{lat}}$, $\mathrm {d_{inter}}$ with increasing $\mathrm {k_r}$ (*left*) and increasing $\mathrm {k_x}$ (*right*) (from *light* to *dark blue* in both figures). The asymptotic conductance of an unbranched root system is given by the *horizontal dashed lines*

Figure 7(left) shows the recurrence that must be solved to obtain the asymptotic conductance of our system $\mathrm {K_{rs,n_{lat}}}$ after addition of $\mathrm {n_{lat}}$ laterals. $\mathrm {k_r}$ and $\mathrm {k_x}$ are the radial conductivity and intrinsic axial conductance of the main root, $\mathrm {d_{inter}}$ and $\mathrm {K_{lat}}$ are the branching distance and the lateral root conductance, respectively. Using Eq. (16), we obtain the following recurrent series after simplification:

$$\begin{aligned} K_{rs,n_{lat}} = \frac{\kappa \left( K_{lat} cosh(\tau d_{inter}) + sinh(\tau d_{inter}) +K_{rs,n_{lat}-1} cosh(\tau d_{inter}) \right) }{K_{lat} sinh(\tau d_{inter}) + \kappa cosh(\tau d_{inter}) + K_{rs,n_{lat}-1} sinh(\tau d_{inter})} \end{aligned}$$

where $\mathrm {K_{rs,n_{lat}}}$ is the conductance of the whole system after addition of $\mathrm {n_{lat}}$ laterals.

Applying the method developed for the homogeneous single discrete root (see “Appendix 4”), we obtain the convergence values of the recurrent series for the whole root system:

23 $$\begin{aligned} \left\{ \begin{array}{c} \kappa _{+,lat} = \frac{-K_{lat} + \sqrt{K_{lat}^2 + 4\left( \kappa ^2 +\frac{K_{lat} \kappa }{tanh(\tau d_{inter})}\right) }}{2} > 0\\ \kappa _{-,lat} = \frac{-K_{lat} - \sqrt{K_{lat}^2 + 4\left( \kappa ^2 +\frac{K_{lat} \kappa }{tanh(\tau d_{inter})}\right) }}{2} < 0 \end{array} \right. \end{aligned}$$

$\mathrm {\kappa _{+,lat}}$ is the asymptotic conductance of the system. This is the root system conductance including the effect of laterals. Interestingly, it yields:

$$\begin{aligned} \kappa _{+,lat} \left\{ \begin{array}{l} \overset{K_{lat} \gg \kappa }{\xrightarrow {}} \frac{\kappa }{tanh(\tau d_{inter})} \\ \overset{K_{lat} \ll \kappa }{\xrightarrow {}} \kappa \\ \overset{d_{inter} \rightarrow 0}{\xrightarrow {}} \sqrt{\frac{K_{lat} k_x}{d_{inter}}} \\ \overset{d_{inter} \rightarrow \infty }{\xrightarrow {}} \kappa \end{array} \right. \end{aligned}$$

In others words, the asymptotic conductance of a RS with laterals depends on four parameters: the radial and axial conductivities of the principal root segments, the conductance of the laterals and the distance between two laterals. When this distance becomes infinite—i.e. there is no more laterals—we come back to the conductance we found in Sect. 2.2 (continuous solution of the single homogeneous root). Same observation if the laterals conductance becomes negligible. Other possible simplification: if the $\mathrm {K_{lat}}$ is much larger than $\mathrm {\kappa }$, the root system conductance is the principal root conductance $\mathrm {\kappa }$ increased by a factor $\mathrm {\frac{1}{tanh(\tau d_{inter})}}$. If the internodal distance becomes very short the asymptotic conductance of the RS depends only on the principal axial conductance, the distance between the successive laterals and their conductance. The fact that a maximal conductance for a root system with laterals is progressively reached as the principal root branch grows puts the basis of allometric relations (links functional—structural properties) for root systems.

The RS conductance has the same shape than the discrete solution for a homogeneous root:

24 $$\begin{aligned} K_{rs,n_{lat}} = \frac{\left( \chi _{lat}\right) ^{n_{lat}} - 1 }{\frac{1}{\kappa _{-,lat}} \left( \chi _{lat}\right) ^{n_{lat}} -\frac{1}{\kappa _{+,lat}}} \end{aligned}$$

with:

25 $$\begin{aligned} \chi _{lat} = \left( \frac{\kappa }{\kappa cosh(\tau d_{inter})- \kappa _{-,lat} sinh(\tau d_{inter})} \right) ^2 \end{aligned}$$

Let us note that in their original paper Landsberg and Fowkes found a similar relation for the total conductance of a root bearing laterals but they considered the following simplification: the laterals were not accounted directly for but their presence was simulated increasing the radial conductivity of the main root everywhere. This assumption leads to overestimate the root system conductance especially when the density of the laterals is low or when their conductance is high. Moreover it does not predict correctly the water uptake location (which is ’diluted’ along the root branch).

In addition this model can be coupled with the previous one (root with heterogeneous properties) to take into account the differentiated zones close to the root tip or at the root basis.

From this solution we can derive how many segments are necessary to reach a certain fraction of the maximal conductance $\mathrm {\kappa _{lat,+}}$. We derive the fraction $\mathrm {\alpha _{lat}}$ of the actual system conductance to the asymptotic one and its inverse function:

26 $$\begin{aligned} \alpha _{lat} (n_{lat}) = \frac{\chi _{lat}^{n_{lat}} - 1}{\frac{\kappa _{+,lat}}{\kappa _{-,lat}}\chi _{lat}^{n_{lat}} - 1} \iff n_{lat}(\alpha _{lat}) = \frac{log\left( \frac{\alpha _{lat} - 1}{\alpha _{lat} \frac{\kappa _{+,lat}}{ \kappa _{-,lat}} - 1 }\right) }{log\left( \chi _{lat} \right) } \end{aligned}$$

Figure 7 shows the asymptotic conductance $\mathrm {\kappa _{+,lat}}$ of the root with laterals, clarifying the effect of $\mathrm {k_{x}}$, $\mathrm {k_{r}}$, $\mathrm {K_{lat}}$, and $\mathrm {d_{inter}}$. The horizontal dashed lines give the asymptotic conductance $\mathrm {\kappa }$ for the principal root without laterals, for different ratio of radial to axial conductances (Eq. 24), increasing the radial conductivity (left, from light to dark blue lines) or axial conductivity (right, from light to dark blue lines). Besides it prevents the accurate location of the root water uptake along the root since the effect of laterals is diluted along the whole root length.

The continuous lines give the conductance of a branched root when $\mathrm {K_{lat}}$ increases and $\mathrm {d_{inter}}$ is 1 cm (open triangles) or 5 cm (open circles). The larger the distance between branches, the more the asymptotic conductance is controlled by the principal root properties. The equivalent conductance starts to be affected by the conductance of the lateral roots after a certain $\mathrm {K_{lat}}$ value that varies with $\mathrm {k_{r}}$. When $\mathrm {K_{lat}}$ tends to zero, the solution becomes equivalent to a single unbranched root system and reaches the dashed lines asymptotically.

Let us note that if the internodal distance between successive laterals becomes very short, the root conductance, as suggested by Landsberg and Fowkes (1978), is given by:

$$\begin{aligned} K_{rs} = \kappa _{new} tanh(\tau _{new} l_{root}) \end{aligned}$$

with the same notation as before and:

$$\begin{aligned} k_{r,new}= & {} k_r + \frac{K_{lat}}{2\pi r d_{inter}} \\ k_{x,new}= & {} k_x \end{aligned}$$

## Applications

In this section, we present possible applications of these models. We first use experimental data published by Zwieniecki et al. (2002) to estimate local root hydraulic properties from multiple global conductance measurements. Then we perform a sensitivity analysis of a maize root system to understand the key factors of its hydraulics. Finally we develop applications regarding quantitative ideotypes.

**Fig. 8:**
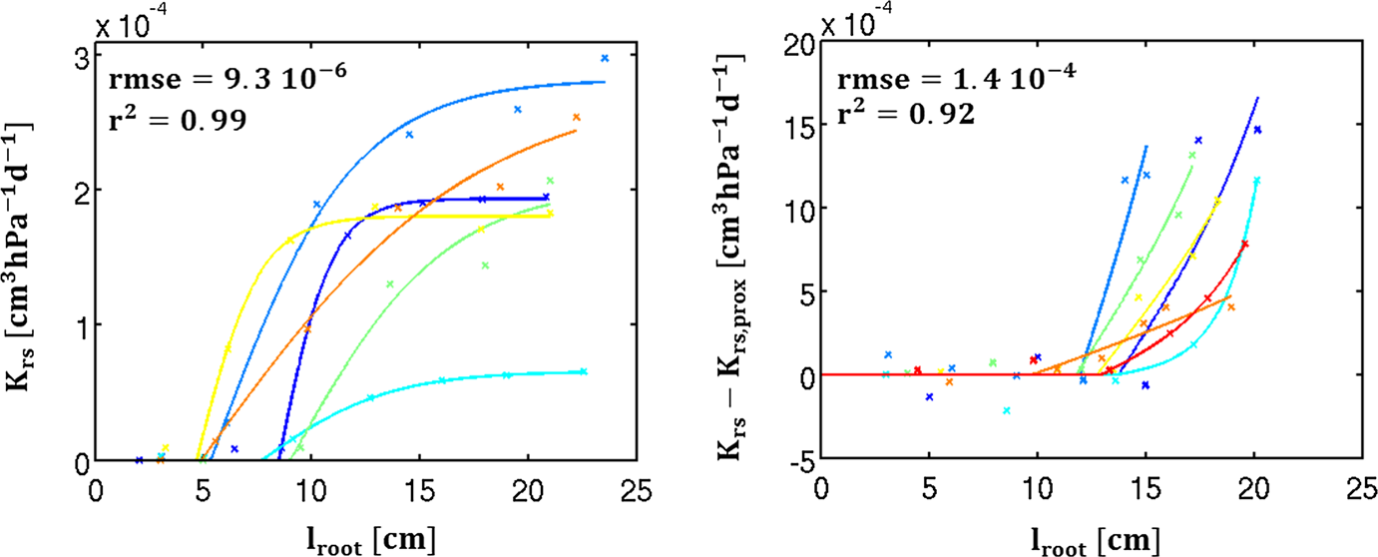
Application of the model to retrieve root local properties (experiments from Zwieniecki et al. 2002): experimental results (*symbols*, each *colour* stand for one particular root) as compared to model best fit (*solid lines*, with the corresponding colours) for both experiments (*left panel* proximal end cut, *right panel* distal end cut)

### Application to experimental data

In a first experiment, Zwieniecki et al. (2002) cut the proximal end of primary maize root branches and measured the conductance of the remaining part. Repeating this procedure several times and for several root axes, they obtained data of water flow under a given potential difference as a function of the distance to the root tip. The experimental results are shown in Fig. 8 (left panel, symbols). Each colour stands for a different root branch analysed by Zwieniecki et al. (2002). A second experiment was performed on other RS in which the distal part of the roots that was cut (right panel, symbols). This time they measured the conductance of the proximal root branch region. Adding some microscopic cross sections to this framework, they observed two distinct zones in the root branches: the axially nonconductive tip and a second radially active zone whose cortex was composed by early metaxylem vessels. Although the root axis is composed of two parts, we can consider that only the second part takes up water and treat the RS as a homogeneous root made only by the oldest segments. Equations from single homogeneous root section are consequently valid. The second equality of Eq. (8) relates the root branch conductance $\mathrm {K_{rs}}$ to the root segment length $\mathrm {l_{root}}$. It can be used directly for the first experiment consequently. This relation was fitted to the dataset in order to optimize local axial and radial conductivity for each root using the reference potential differences mentioned in the study of 0.38 MPa. For the second experiment the measured flow are given as the difference between the total uncut root flow and the flow of the proximal end. So we present the results in a similar form: $\mathrm {K_{rs} (l_{root}) - K_{rs,prox}}$ with $K_{rs,prox} = \kappa tanh(\tau z)$.

Only considering one root zone allows an extremely good fit to the observations represented by the colour symbols. We obtain a very strong correlation between the modelled (solid lines) and the measured conductances (symbols): $\mathrm {r^2=0.99}$ as shown in the left panel representing the first experiment. The second experiment (right panel) provides results almost as good as the previous ones: $\mathrm {r^2=0.92}$. In this plot, the results are presented in a form similar to the the original one of Zwieniecki et al. (2002): as the difference of the total root conductance minus the conductance of the proximal zone. As it can be seen easily from the left panel, as the root grows (but this is valid also for the second experiment), the conductance reaches a plateau just as the Eq. (8) predicts it. It means that the xylem conductance becomes limiting the water flow in these roots.

### Application example: a maize root system

**Fig. 9:**
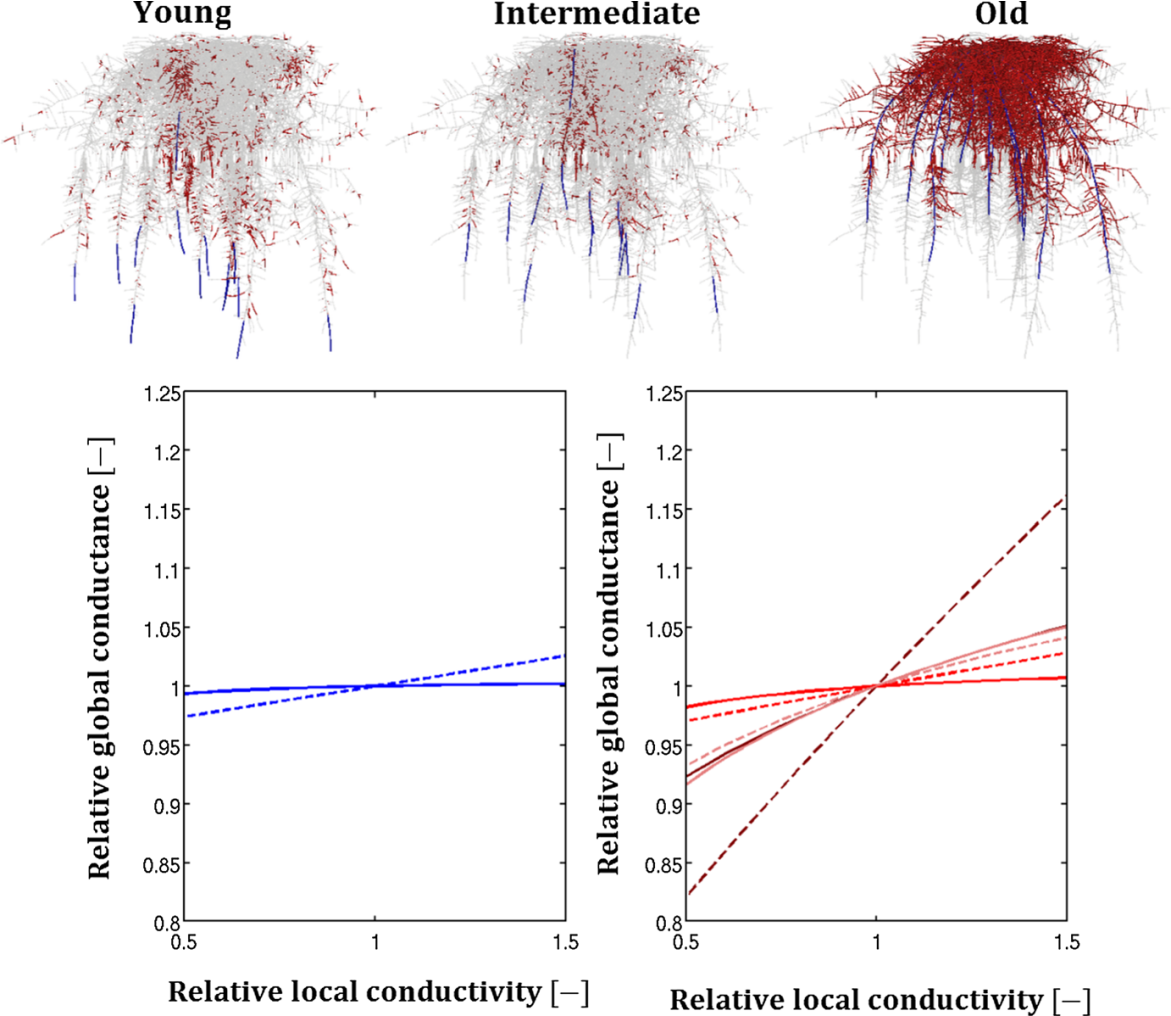
Sensitivity analysis of a maize root system: the top panels represent a maize root system architecture highlighting the different root zones: young (*left*), intermediate (*center*) and old (*right*) root zones for both primaries (*blue*) and lateral (*red*) roots. *Bottom* subplots show the effect of modifying local radial (*dashed*) or axial (*solid*) conductivities of the primary (*left*) or lateral (*right*) roots on the RS conductance in a one-by-one sensitivity analysis. The *curves* are coloured as a function of the segment ages and consequently their hydraulic conductivities: the darker, the younger

The functions developed in the theory are of potential use for developing quantitative ideotypes, i.e., quantitative estimatse of optimal root hydraulic or architectural traits for drought stress tolerance. We consider as an example a maize RS generated by RootTyp (Pags et al. 2004). This RS is made of several primary roots attached to the basis of a stem. On these parallel primaries are attached regularly spaced laterals. We used the relationships of Doussan et al. (1998b) (see Figure 4) to represent the evolution of root segment radial and axial hydraulic properties with root segment age as a succession of plateau for both primaries and laterals. The conductance of a mature maize lateral root (whose oldest segments have the hydraulic properties of the last plateau) can be predicted using the equations of the root branch with heteregeneous properties section: we used an equation similar to Eq. (16) with three successive plateau’s. To derive the total RS conductance we assume that the stem is not taking up water (its radial conductivity is zero) and that its xylem conductance is hydraulically non-limiting (its axial conductivity is very high). Consequently the RS conductance is simply the sum of the primary conductances. As illustrated in the top panel of Fig. 9 the majority of the root primary surface for a mature maize RS is made by the oldest zone so that we can consider with good approximation the primary root hydraulic properties as uniform when analysing a mature root system. Around 70% of the root surface of the primaries is made of segments older than 23 days, 18% have between 10 and 25 days, the remaining 12% is made of young segments. (younger than 10 days) Similar contribution is observed for the root surface of the laterals (14% younger, 16% intermediate and 66% older, respectively, the rest is the non-conductive tips).

We can use the model including laterals to calculate the primary conductance and finally the RS conductance. The $\mathrm {d_{inter}}$ parameter is provided by the mean value of the inter-branches distance of RootTyp parametrized for maize (Couvreur et al. 2014).

In the bottom panels of Fig. 9 we consider a change of $\pm 50$% for the local conductivities of both primary (left) and lateral (right) roots, everything else kept constant. In these panels the dashed lines stand for the radial conductivity while the solid lines represent the axial intrinsic conductances. The colour blue indicate the primary roots and the red colour, the laterals. The darker the lines, the younger the zones.

The interest of such an example is to evaluate the potential benefit of increasing locally the root conductivity on RS conductance.Performing a sensitivity analysis on mature roots, we investigate which zone is the most resistive part of complex root systems. This sensitivity analysis is based on both hydraulic (obtained by Doussan et al. 1998b) and architectural parameters (growth and shape parameters of the maize plant for RootTyp (Pags et al. 2004).

We can first notice that hydraulic property relative changes of primaries would not have a big impact on the total RS conductance. Indeed the axial conductivity of mature primaries are high enough to conduct the total amount of water until the collar while their radial conductivity is so low that even if the pressure head drops until the collar would be negligible increasing them the consequent change in conductance would be tiny.

The radial conductivity of young segments of lateral roots will have an important (the biggest) impact on global conductance even if they are not the majority of lateral segments as it can be seen from the left top panel (they constitue only 14% of the lateral root surfaces).

Javot and Maurel (2002) reviewed the aquaporin expression in roots and its effect on the root hydraulic conductivity. They listed a set of examples demonstrating the possible regulation of the root conductance according to outer stimuli. Using the present model and this sensitivity analysis we can predict the magnitude of a change in local radial conductivity needed to induce a desired modification of the water uptake in a given soil (so far only in homogeneous soil conditions but the present model could and will be coupled in the future with a soil model to simulate transient flow). Another application would be the effect of drought period and its consequent root cells shrinkage leading to a decrease of the soil–root contact and thus of the root radial conductance on macroscopic behaviour (North and Nobel 1991). Finally, the benefits of increasing local xylem vessels (Vercambre et al. 2002) or rise the radial conductivity of certain zones (Blum 2010) can be predicted using both this approach and/or complete simulations (Leitner et al. 2014b). For example from the picture (Fig. 9) it can readily be seen that increasing axial conductances of both young and intermediate lateral segments would allow raising the RS conductance significantly. Other architectural parameters can also be easily tested with this model. For example increasing the root density by reducing the value of $\mathrm {d_{inter}}$.

If we performed here a one-by-one sensitivity analysis, combination of traits may also be considered. RS ideotypes as suggested by Lynch (2013) for maize may be evaluated through the here-developed models if proposed under the form of quantitative traits.

### Allometric relations as a function of hydraulic properties

Allometry is the study of the relationship of body size to shape, anatomy, physiology and finally behaviour (Damuth 2001). For root systems allemoteric relations have already been proposed by Biondini (2008) linking structural functions to local functional properties.

It has been demonstrated in the section theory (see single homogeneous root or root system with laterals) that a maximal value of $\mathrm {K_{rs}}$ is progressively approached as the root length increases. This means that an optimal root length must exist in terms of carbon cost as compared to root conductance gain. This optimal length allows defining allometric relations between structural and functional root properties. Here below we define these relations for 2 cases.

First we consider a single homogeneous branch and ask the following question: If we increase the radial (resp. axial) conductivity of a homogeneous root by a factor a (resp. by a factor of b), how much may we reduce the root length and keep the same global conductance value?

Mathematically, this question can be addressed by finding the length $\mathrm {l_{root}^*}$ that keeps the same global conductance while its local hydraulic properties are altered.

$$\begin{aligned} l_{root}^* \qquad \text {such as} \qquad K_{rs}(l_{root}) = \left\{ \begin{array}{l} \sqrt{2 \pi r a k_r k_x} tanh\left( \sqrt{\frac{2 \pi r a k_r}{k_x} l_{root}^*}\right) \\ \sqrt{2 \pi r k_r b k_x} tanh\left( \sqrt{\frac{2 \pi r k_r}{b k_x} l_{root}^*}\right) \\ \end{array} \right. \end{aligned}$$

If we take as reference global conductance value half of the maximal original conductance $K_{rs}(l_{root}) = \frac{\kappa }{2}$. Using Eq. (8) we obtain

27 $$\begin{aligned} \left\{ \begin{array}{l} \frac{l_{root}^*}{l_{root}} = \frac{1}{\sqrt{a}} \frac{atanh\left( \frac{1}{2} \frac{1}{\sqrt{a}}\right) }{atanh\left( \frac{1}{2} \right) }\\ \frac{l_{root}^*}{l_{root}} = {\sqrt{b}}\frac{atanh\left( \frac{1}{2} \frac{1}{\sqrt{b}}\right) }{atanh\left( \frac{1}{2} \right) }\end{array} \right. \end{aligned}$$

**Fig. 10:**
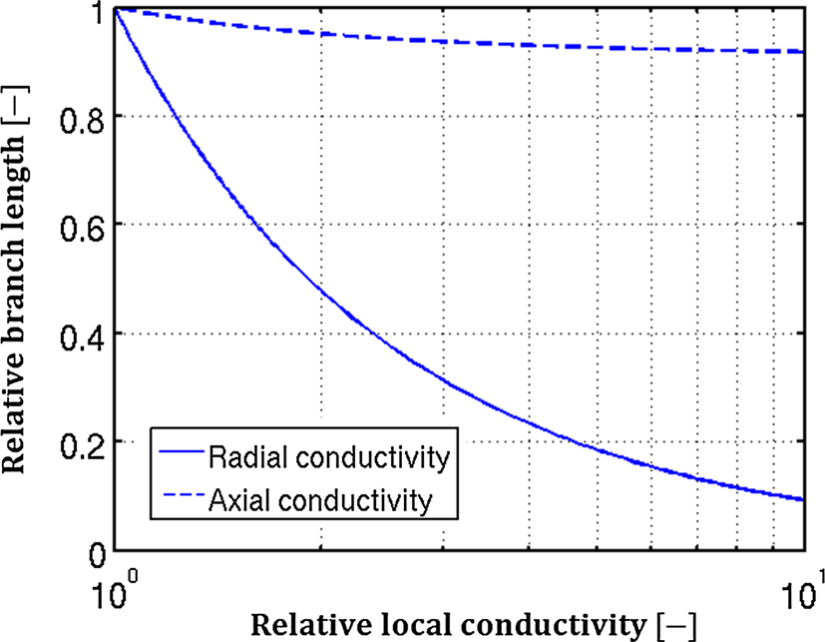
Quantitative phenotyping illustration: ratio of new to old root branch length needed to reach half the original asymptotic conductance when increasing the radial conductivity by a factor a (*solid line*) or the axial conductivity when increasing the axial conductivity by a factor b (*dashed line*). The horizontal axis represents the factor a or b

Interestingly Eqs. (27) are independent on the root hydraulics or geometric properties. Increasing the radial (resp. axial) root branch conductivity, Eq. (27) a (resp. b) provides the new length necessary to reach half of the original asymptotic conductance normalized by the previous length. Figure 10 shows these functions when the factor $\mathrm {a}$ (solid line) or $\mathrm {b}$ are in the range [1 10]. The new root branch length is always smaller when increasing the radial conductivity as compared to increasing the axial conductivity. Moreover the new branch length is always larger than 0.9 in this range when raising the axial hydraulic properties while it decreases until 0.1 when focusing on the radial hydraulic properties.

The second example shown in this section deals with RS including laterals. Equation (23) gives the maximal conductance of a branched root system as a function of lateral conductance, local conductivities of the main root and the internodal distance. If the internodal distance increases or the lateral conductance decreases, the asymptotic RS conductance will progressively converge towards $\mathrm {\kappa }$ the asymptotic conductance of an unbranched root branch. Beyond a maximal branching length or a minimal lateral conductance, the lateral roots have no more effects on the RS global conductance. Thus for a set of three paramaters ($\mathrm {k_r}$, $\mathrm {k_x}$ and $\mathrm {d_{inter}}$ or $\mathrm {K_{lat}}$) a fourth one can be derived that enables increasing the unbranched conductance by a fraction $\mathrm {\epsilon \, [-]}$. Mathematically we are looking for the minimal $\mathrm {K_{lat}^*}$ or maximal $\mathrm {d_{inter}^*}$ such as:

28 $$\begin{aligned} \left( 1 + \epsilon \right) \kappa = \kappa _{+,lat} \end{aligned}$$

with $$\mathrm {\epsilon > 0}$$.

**Fig. 11:**
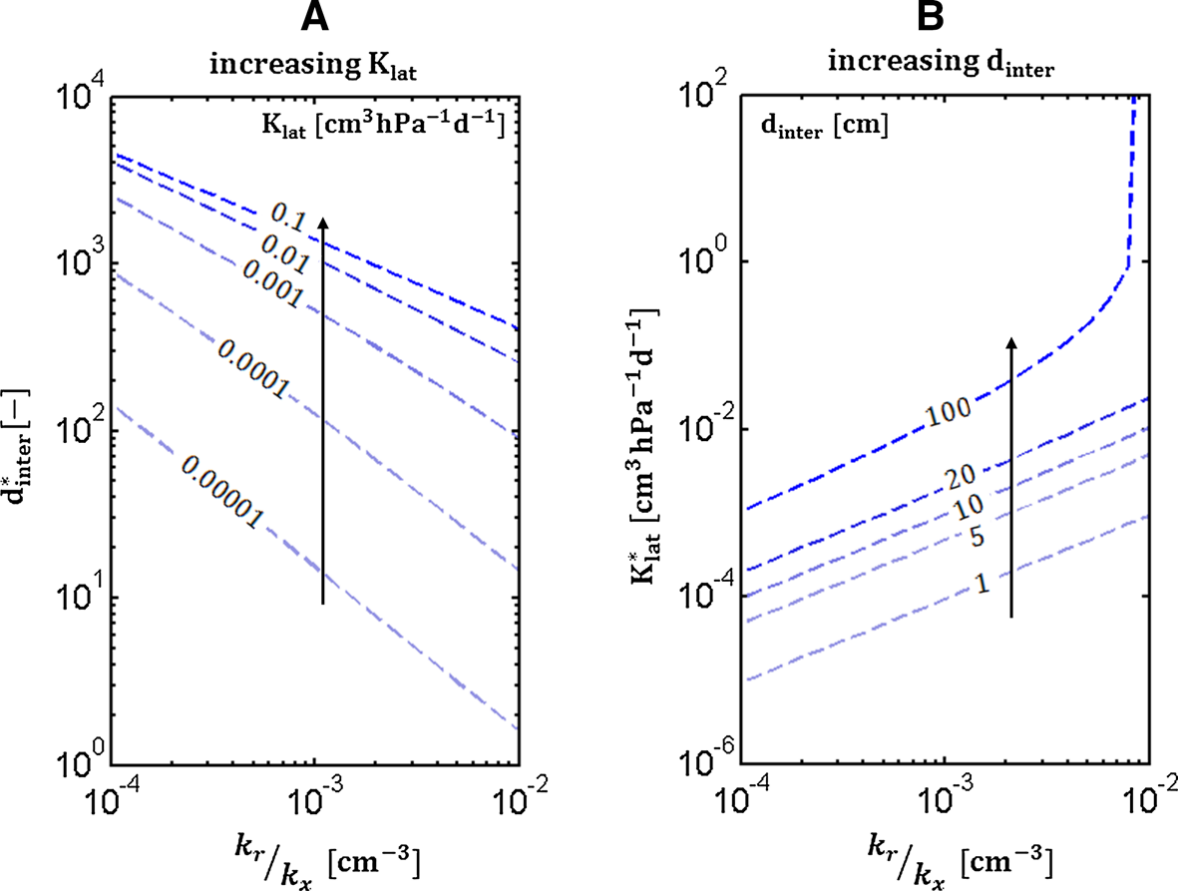
Example of relationship between architecture and hydraulics: maximal internodal distance $\mathrm {d_{inter}^*}$ (*left*) and minimal $\mathrm {K_{lat}}^*$ (*right*) that allows increasing the RS asymptotic conductance by 5% as compared to an unbranched root branch as a function of $\mathrm {\frac{k_r}{k_x}}$ ratio of the main root

Here again an analytical solution of the problem can be found. In Fig. 11, we plot these relations as a function of $\mathrm {k_r}$ to $\mathrm {k_x}$ ratio. In the left subplot, five different levels of $\mathrm {K_{lat}}$ are considered while in the right subplot we draw the results for four distinct internodal distances. In other words, if we consider a plant root whose $\mathrm {\frac{k_r}{k_x}}$ ratio is fixed as well as $\mathrm {K_{lat}}$, then Fig. 11a gives the maximal internodal distance that makes sense in a hydraulic framework that increases significantly the unbranched conductance). Beyond this maximal internodal distance the primary axial intrinsic conductance avoids a significant contribution of the laterals on the RS conductance. The same explanation is valid for the second subplot: if $\mathrm {\frac{k_r}{k_x}}$ and $\mathrm {d_{inter}}$ are fixed then the five lines represent the minimal conductance of laterals compatible with a significant increase of the global conductance. $\mathrm {\epsilon }$ was fixed to 5%. If the lateral conductance is lower, it is too tiny compared to the primary radial conductivity to significantly contribute to the RS conductance.

### Future model applications

The emergence of common database collecting root structural, topological and hydraulic properties in a unique format (Lobet et al. 2015) makes possible the use of the present model to calculate and to collect macroscopic parameters of any listed species.

A coupling can also be made between any software analysing root architecture from experiments (a non-exhaustive list is referenced by Lobet et al. 2013) and such a model enabling an automatized analysis of global properties of genotypes if local conductivity functions are available a priori. Otherwise an additional work of determining these functions will be needed but the equations presented in this paper can also be useful to retrieve shapes and values of these conductivity relationships from some global conductance measurements added to any technique delivering a complete root architecture (topology, age and order distribution such as in Leitner et al. 2014a)

Finally this model could be coupled with the novel macroscopic root water uptake model developed by Couvreur et al. (2012) to investigate the effect of modifying any trait first on plant parameters and afterwards on plant ability to maintain or to increase its yield (through transpiration) under drought condition when the environment is provided. In further studies, breeding proposals of Comas et al. (2013), Lynch (2013) and Wasson et al. (2012) will be tested in silico in various contexts defined as a combination of soil and climate. Using a model solving water flow in soil and plant will also allow us to retrieve the impact of given traits on soil moisture and potential distribution (Teuling et al. 2006). The new developments presented here allow us to calculate plant-scale parameters of idealized root systems. These parameters (calculated in homogeneous conditions) can be used for simulating root water uptake in non-homogeneous conditions as demonstrated by Couvreur et al. (2012). The methodology presented here constitutes thus the first step in a larger study to predict the plant performance in contrasted (and heterogeneous) environments. Our results are tools to accurately and efficiently calculate plant-scale parameters that can be used in turn in soil–root system model simulating the water flow to assess plant performance. They consequently need to be coupled with soil model (de Jong van Lier et al. 2008). The performance of root systems when simulated in contrasted environment will be indeed highly dependent on the macroscopic parameters (that represent the ability to take up water and the location of the root water uptake) as well as the pedoclimatic situations. Next studies will consequently focus on plant–environment interactions in order to look for best associations and to decipher key traits for an optimal root water uptake in contrasted and heterogeneous conditions.

Some of the major roles of root systems are water and nutrient uptake from soils. In this paper, we considered the hydraulic macroscopic parameters of the root system and their dependence on the hydraulic properties of root segments and the distribution of lateral roots. However, the search for an optimized root system is a multi-objective optimization problem that should consider optimal water but also nutrient uptake under minimal carbon costs. This optimum might furthermore depend on the soil hydraulic and chemical properties, the nutrient and water distribution and the climate. As far as the nutrient uptake is considered, further work is needed to link the distribution of nutrient uptake to root properties and nutrient distributions. But, the current work partly contributes to this issue since water uptake distributions influence nutrient transport to the root surfaces directly by advective flow and indirectly by changing water content and consequently the diffusion coefficient. Next studies simulating simultaneous water and nutrient uptakes may help identifying key properties for the capture of these resources in each type of environments.

Further studies will consequently focus on the use of such equations to decipher the soil–plant relation in a complete Root–Soil model solving the water flow in soil and root systems in three dimensions and in particular on the relations between the macroscopic parameters and the cumulative transpiration in a range of environments.

## Conclusion

We present novel mathematical functions that predict plant scale hydraulic properties from measurable local structural and functional properties. The first parameter is the root global conductance $\mathrm {K_{rs}}$; the second parameter describes the potential relative uptake in uniform soil and is called the Standard Uptake Density $\mathrm {SUD}$. These new root models were developed based on analytical solutions (and their approximations) of root water flow equations. Such functions may allow breeders to quantitatively a priori assess how a local root trait affects the global plant hydraulic properties, which opens new avenues for optimizing plant drougth tolerance for instance.

Three types of simplistic root systems were considered: a homogeneous single root branch, a single root branch split into several homogeneous zones or a single root branch with regularly spaced laterals. For these three root systems, macroscopic parameters can always be predicted as a function of local functional and architectural root traits. The relevant architectural traits were the total root length, the length of the homogeneous root zone, the distance between laterals. The functional parameters were the axial conductance and the radial conductivity of the homogeneous zones, of the primary and of the laterals.

For a homogeneous root or a root with laterals, it was demonstrated that a maximum conductance is reached after a certain length for given set of $k_r$ and $k_x$ values, which mean that optimal root lengths must exist in terms of hydraulics.

These models were successfully applied to laboratory data or used in sensitivity analyses of RHA to investigate the impact of local traits on global plant hydraulic behaviour. Finally they can be seen as a tool for deriving quantitative links between hydraulic and architectural traits of root systems.

Based on these simple RS functions, models for more realistic RS architectures can be built, paving the way for quantitative estimation of the impact of changes in root traits on plant uptake efficiency. It is expected that other architectural traits like root angles will play a role in the estimate of $\mathrm {SUD}$ with more complex systems.

Applications of these functions may allow one to quantitatively assess *in silico* ideotypes, i.e. root systems with ideal traits for drought tolerance as proposed by Comas et al. (2013), Lynch (2013) and Wasson et al. (2012).

## Appendix 1

**Table:**

Symbol	Dimension	Description
$\alpha$	−	Fraction of the asymptotic conductance reached by root length $l^*$
$\alpha _{lat}$	$L^3 P^{-1} T^{-1}$	Fraction of the asymptotic conductance reached with ${n_{lat}}$
$\chi$	−	Eq. (22)
$\chi _{lat}$	$L^3 P^{-1} T^{-1}$	Eq. (25)
$d_{inter}$	*L*	Inter-branching distance
$H_{collar}$	*P*	Collar water potential
$H_{s,eq}$	*P*	Equivalent soil water potential
$H_{soil}$	*P*	Soil water potential
$H_{sr}$	*P*	Soil–root water potential
$H_x$	*P*	Xylem water potential
*i*	−	Root zone number
$J_r$	$L^3 T^{-1}$	Radial volumetric flow rate
$J_x$	$L^3 T^{-1}$	Axial volumetric flow rate
$\kappa$	$L^3 P^{-1} T^{-1}$	Asymptotic root conductance
$\kappa _+$	$L^3 P^{-1} T^{-1}$	Eq. (21)
$\kappa _-$	$L^3 P^{-1} T^{-1}$	Eq. (21)
$\kappa _{+,lat}$	$L^3 P^{-1} T^{-1}$	Eq. (23)
$\kappa _{-,lat}$	$L^3 P^{-1} T^{-1}$	Eq. (23)
$K_{comp}$	$L^3 P^{-1} T^{-1}$	Compensatory water uptake conductance
$K_{rs}$	$L^3 P^{-1} T^{-1}$	Global conductance of the root system
$K_{rs,n_{lat}}$	$L^3 P^{-1} T^{-1}$	Root system conductance after addition of ${n_{lat}}$ laterals
$k_r$	$L P^{-1} T^{-1}$	Root radial conductivity
$k_x$	$L^4 P^{-1} T^{-1}$	Root axial conductivity
$K_r$	$L^3 P^{-1} T^{-1}$	Segment radial conductance
$K_x$	$L^3 P^{-1} T^{-1}$	Segment axial conductance
$l^*$	*L*	Root length for a specific for a faction $\alpha$ of the asymptotic conductance
$l_{root}$	*L*	Root length
$l_{seg}$	*L*	Segment length
$N_{inter}$	−	Number of root segments between two successive branches
*n*	−	Root segment number
${n_{lat}}$	−	Number of laterals
$q_r$	$L T^{-1}$	Root radial flux
*r*	*L*	Root radius
*SUD*	$L^{-1}$	Standard uptake density
*SUF*	−	Standard uptake fraction
$\tau$	$T^{-1}$	Root intrinsic property
$T_{act}$	$L^3 T^{-1}$	Actual transpiration of the root system
*z*	*L*	Distance to root tip

## Appendix 2

The axial flow $\mathrm {J_x \, \left[ L^3 T^{-1}\right] }$ inside a homogeneous root is given by:

29 $$\begin{aligned} J_x(z, l_{root})=-k_x\left( \frac{d H_x(z, l_{root})}{d z}\right) \end{aligned}$$

This flow is a function of the position inside the root $\mathrm {z \, [L]}$ and the root length. In Eq. (29), $\mathrm {H_x \, [P]}$ is the total water potential (the sum of gravitational and pressure potentials). Mass conservation for a segment of length $\mathrm {dz \, [L]}$ (see Fig. 1a) leads to:

30 $$\begin{aligned} -2\pi r k_r\left( H_x(z, l_{root})-H_{soil}\right) = \frac{d J_x(z, l_{root})}{d z} \end{aligned}$$

The left hand side of the previous equation gives the radial flow between the soil–root interface and the inner root, $\mathrm {[L^3 T^{-1}]}$ divided by a root layer $\mathrm {dz \, [L]}$. Combining Eqs. (29) and (30) we obtain a differential equation:

$$\begin{aligned} -2\pi r k_r\left( H_x(z, l_{root})-H_{soil}\right) = - k_x\frac{d^2 H_x(z, l_{root})}{d z^2} \end{aligned}$$

The general solution of this partial derivative equation has the shape:

$$\begin{aligned} H_x (z, l_{root})=H_{soil} + c_1 exp\left( \tau z\right) +c_2 exp\left( -\tau z\right) \end{aligned}$$

with: $$\mathrm {\tau = \sqrt{\frac{2 \pi r k_r}{k_x}} \, [L^{-1}]}$$.

If we impose the following boundary conditions $\mathrm {J_x\left( z = 0,l_{root}\right) = 0}$ and $\mathrm {H}_\mathrm {x}\big (\mathrm {z} = \mathrm {l}_{\mathrm {root}}$, $\mathrm {l}_{\mathrm {root}}\big ) = \mathrm {H}_{\mathrm {collar}}$, we obtain the unique solution for the water potential inside the root:

31 $$\begin{aligned} H_x (z, l_{root})= H_{soil}+ \left( {H_{collar}-H_{soil}}\right) \frac{cosh\left( \tau z\right) }{cosh\left( \tau l\right) } \end{aligned}$$

Using Eqs. (29) and (30) and the specific solution (31) we may find the axial and radial flows:

$$\begin{aligned} J_x (z, l_{root})= & {} k_x \tau \left( {H_{soil} - H_{collar}}\right) \frac{sinh\left( \tau z\right) }{cosh\left( \tau l\right) }\\ J_r (z, l_{root})= & {} dz k_x \tau ^2 \left( {H_{soil}-H_{collar}}\right) \frac{cosh\left( \tau z\right) }{cosh\left( \tau l\right) } \end{aligned}$$

## Appendix 3

To derive the discrete recursion formula from discretising the continuum equations we consider a homogeneous root of length $\mathrm {l_{root}}$ and made of n segments of length $\mathrm {l_{seg}}$ in a homogeneous of water potential $H_{soil}$. We start from the conductance definition: the root branch conductance is the axial flow divided by the difference of water potential

$$\begin{aligned} \frac{1}{K_{rs,n}} = \frac{H_{soil} - H_{x,n}}{J_{x,n}} \end{aligned}$$

It is equivalent to:

$$\begin{aligned} \frac{1}{K_{rs,n}} = \frac{H_{soil} - H_{x,n-1} + H_{x,n-1} - H_{x,n}}{J_{x,n}} \end{aligned}$$

Splitting into two terms the right hand side, it yields:

$$\begin{aligned} \frac{1}{K_{rs,n}} = \frac{H_{soil} - H_{x,n-1}}{J_{x,n}} + \frac{ H_{x,n-1} - H_{x,n}}{J_{x,n}} \end{aligned}$$

The first term can be expressed as the root conductance when one segment is removed. The second term is simply the inverse of the segment axial conductance:

32 $$\begin{aligned} \frac{1}{K_{rs,n}} = \frac{1}{K_{rs,n-1}} \frac{J_{x,n-1}}{J_x} + \frac{l_{seg}}{k_x} \end{aligned}$$

On the other hand, the water flow entering the root inside the last root segment is:

$$\begin{aligned} J_{x,n} - J_{x,n-1} = 2 \pi r k_r \left( H_{soil} - H_{x,n-1} \right) l_{seg} \end{aligned}$$

Dividing by $$\mathrm {J_{x,n-1}}$$:

$$\begin{aligned} \frac{J_{x,n}}{J_{x,n-1}} - 1 = \frac{2 \pi r k_r \left( H_{soil} - H_{x,n-1} \right) l_{seg}}{J_{x,n-1}} \end{aligned}$$

We obtain

33 $$\begin{aligned} \frac{J_{x,n}}{J_{x,n-1}} = \frac{2 \pi r k_r l_{seg}}{K_{rs,n-1}} + 1 \end{aligned}$$

Finally combing Eqs. (32) and (33), it yields:

$$\begin{aligned} \frac{1}{K_{rs,n}} = \frac{l_{seg}}{k_x} + \frac{1}{2 \pi r k_r l_{seg} + K_{rs,n-1}} \end{aligned}$$

Which is equivalent to:

$$\begin{aligned} \frac{1}{K_{rs,n}} = \frac{1}{K_x} + \frac{1}{ K_r + K_{rs,n-1}} \end{aligned}$$

## Appendix 4

We want to solve the recurrent series shown in Fig. 6 to derive the root conductance $\mathrm {K_{rs,n}}$ after addition of n identical segments. It can be demonstrated that this sequence converges and the possible values of convergence ($\mathrm {\kappa _-}$ and $\mathrm {\kappa _+}$) can be expressed as follows:

34 $$\begin{aligned} \frac{1}{K_{rs}} = {\frac{1}{K_{x}}+\frac{1}{K_r + K_{rs}}} \iff \begin{array} {l} \kappa _- = \frac{-K_r-\sqrt{K^2_r+4K_rK_x}}{2} <0 \\ \kappa _+=\frac{-K_r+\sqrt{K^2_r+4K_rK_x}}{2} >0 \end{array} \end{aligned}$$

These solutions are found assuming $\mathrm {K_{rs,n} = K_{rs,n-1}}$. As conductances are always larger than zero, $\mathrm {\kappa _+}$ is the only possible solution.

We want to discover now how fast the addition of new resistances will make the model converge to this value. To do so we can rewrite equation Eq. (34) as the following sequence whose $\mathrm {K_{rs,n}}$ is the nth element:

$$\begin{aligned} K_{rs,n+1}=f(K_{rs,n})= \frac{1}{\frac{1}{K_{x}}+\frac{1}{K_r + K_{rs,n}}}=\frac{K_x K_{rs,n}+ K_r K_x}{K_{rs,n}+\left( K_r + K_x \right) }=\frac{a K_{rs,n}+b}{c K_{rs,n}+d} \end{aligned}$$

with:

$$\begin{aligned} \,\left\{ \begin{array}{l} f(x)= \left( \frac{1}{K_x} + \frac{1}{K_r + x}\right) ^{-1}\\ a = K_x \\ b =K_r K_x \\ c = 1 \\ d =K_r + K_x \end{array} \right. \end{aligned}$$

Formulated now as an homographic sequence we can evaluate the rate of convergence. This convergence speed indeed is defined by:

$$\begin{aligned} \frac{\mid K_{rs,n+1}-x^{*} \mid }{\mid K_{rs,n}-x^{*} \mid } \end{aligned}$$

where $\mathrm {x^{*}}$ is the value of convergence ($\mathrm { = \kappa _+}$). But in the case of an homographic sequence, this can be simplified and tends to absolute value of the derivative of the function f, $\mathrm {\mid f^{'}(x^{*})\mid }$ :

$$\begin{aligned} \frac{\mid K_{rs,n+1}-x^{*} \mid }{\mid K_{rs,n}-x^{*} \mid } = \frac{\mid f(K_{rs,n})-f(x^{*})\mid }{\mid K_{rs,n}-x^{*}\mid } \rightarrow \mid f^{'}(x^{*}) \mid \end{aligned}$$

The derivative in this case yields:

$$\begin{aligned} \mid f^{'}(x)\mid = \left( \frac{1}{\frac{1}{K_{x}}+\frac{1}{K_r + x}} \right) ^{'} = \frac{K^2_x}{\left( x + K_r + K_x\right) ^{2}} \end{aligned}$$

This function evaluated in $\mathrm {x^{*}=\kappa _+}$ yields:

$$\begin{aligned} \mid f^{'}(x^{*})\mid = \left( \frac{\kappa _+}{\kappa _-}\right) ^2 \end{aligned}$$

The latter equality can be obtained with mathematical manipulations (not shown here). Now we know the speed of convergence, the homographic sequence can be transformed into a geometrical one called hereafter $v_n$:

$$\begin{aligned} v_{n+1}=\left( \frac{\kappa _+}{\kappa _-}\right) ^2 v_n \end{aligned}$$

Link between both series is:

35 $$\begin{aligned} K_{rs,n} = \frac{{v_n}{\kappa _-} - {\kappa _+}}{v_n -1} \end{aligned}$$

The previous steps allow us to predict the global conductance of a root with its number $\mathrm {n}$ of identical segments writing first the following equality

36 $$\begin{aligned} v_{n}= v_1 \left[ \left( \frac{\kappa _+}{\kappa _-}\right) ^{2}\right] ^n= \frac{K_0 - \kappa _+}{K_0 - \kappa _-} \left[ \left( \frac{\kappa _+}{\kappa _-}\right) ^{2}\right] ^n \end{aligned}$$

with $\mathrm {K_0 \, [L^3 T^{-1} T^{-1}]}$ an initial conductance connected the considered root branch. Because the v sequence tends to zero the equivalent resistance will tend towards the positive fixed point, i.e. solution of Eq. (34) when the number of segments is high enough. Finally combining Eqs. (35) and (36) we obtain:

$$\begin{aligned} K_{rs,n}= \frac{{\frac{K_0 - \kappa _+}{K_0 - \kappa _-}\left[ \left( \frac{\kappa _+}{\kappa _-}\right) ^{2}\right] ^n}{\kappa _-} - {\kappa _+}}{\frac{K_0 - \kappa _+}{K_0 - \kappa _-} \left[ \left( \frac{\kappa _+}{\kappa _-}\right) ^{2}\right] ^n -1} \end{aligned}$$

And if $\mathrm {K}_0 = 0$ (i.e. no initial conductance attached to the root branch), we obtain:

$$\begin{aligned} K_{rs,n}= \frac{\left[ \left( \frac{\kappa _+}{\kappa _-}\right) ^{2}\right] ^{n} - 1 }{ \frac{1}{\kappa _-}\left[ \left( \frac{\kappa _+}{\kappa _-}\right) ^{2}\right] ^{n} - \frac{1}{\kappa _+}} \end{aligned}$$

## Appendix 5

To calculate the Standard Uptake Fraction of an homogeneous root, we proceed as follow: the key variable in the SUF determination is the xylem potential of each segment since, in homogeneous condition, the difference between this value and the outside (uniform) potential gives the product of the radial conductance and the lateral flux. Kirchoff and Ohm laws give us two equations (we define now the first segment at the top of the root rather than at the distal end of the root and we consider a saturated soil: $\mathrm {H_{soil} = 0}$ for sake of simplicity):

$$\begin{aligned} \left\{ \begin{array}{l} K_{x,n} \left( H_{x,n} - H_{x,n-1} \right) =J_{x,n}\\ J_{x,n-1}=J_{x,n}-{K_{r,n-1}} {H_{x,n-1}}\\ \end{array} \right. \end{aligned}$$

These equations can be summarised as a system of two recurrent equations:

$$\begin{aligned} \left( \begin{array}{c} H_{x,n}\\ J_{x,n}\end{array}\right) = \left( \begin{array}{c c} 1 + \frac{K_{r,n}}{K_{x,n}} &{} \frac{1}{K_{x,n}} \\ {K_{r,n}} &{}1 \\ \end{array}\right) . \left( \begin{array}{c} H_{x,n-1}\\ J_{x,n-1}\end{array}\right) \end{aligned}$$

At the nth segment of an homogeneous zone ($\mathrm {K_{r,n} = K_r}$ and $\mathrm {K_{x,n} = K_x}$) we can state:

$$\begin{aligned} \left( \begin{array}{c} H_{x,n}\\ J_{x,n}\end{array}\right) = \left( \begin{array}{c c} 1+ \frac{K_{r}}{K_{x}} &{} \frac{1}{K_x} \\ {K_{r}} &{}1 \\ \end{array} \right) ^{n-1} . \left( \begin{array}{c} H_{x,1}\\ J_{x,1}\end{array}\right) \end{aligned}$$

Since we have $\mathrm {K_{rs,n} = \kappa _+ \alpha }$ and $\mathrm {T_{act} = -H_{collar} \kappa _+ \alpha }$ for the global root conductance $\mathrm {K_{rs,n}}$ and for the collar flow $\mathrm {T_{act}}$, respectively.

37 $$\begin{aligned} \left( \begin{array}{c} H_{x,n}\\ J_{x,n}\end{array}\right) = \left( \begin{array}{c c} 1+ \frac{K_{r}}{K_{x}} &{} \frac{1}{K_x} \\ {K_{r}} &{}1 \\ \end{array} \right) ^{n-1} . \left( \begin{array}{c} H_{collar} (1 + \frac{\kappa _{+} \alpha }{K_x})\\ {-H_{collar}}\kappa _+ \alpha \end{array}\right) \end{aligned}$$

If we define matrix $$\mathrm {A = \left( \begin{array}{c c} 1+ \frac{K_{r}}{K_{x}} &{} \frac{1}{K_x} \\ {K_{r}} &{}1 \\ \end{array} \right) } $$ we can, finding the eigenvalues and eigenvectors of $\mathrm {{A}}$, develop the following relations:

$$\begin{aligned} {A}^{n-1}= {P}.{\varLambda }^{n-1}.{P}^{-1} \end{aligned}$$

with:

$$\begin{aligned} P= & {} \left[ \begin{array}{c c} -\frac{\kappa _-}{K_r K_x}&{} -\frac{\kappa _+}{K_r K_x} \\ 1 &{} 1 \\ \end{array} \right] \\ \varLambda= & {} \left[ \begin{array}{c c} 1 - \frac{\kappa _-}{K_x} &{} 0 \\ 0 &{} 1 - \frac{\kappa _+}{K_x} \\ \end{array} \right] \end{aligned}$$

and

$$\begin{aligned} P^{-1} = \left[ \begin{array}{c c}\frac{K_r K_x}{\kappa _+ - \kappa _-} &{}\frac{\kappa _+}{\kappa _+-\kappa _-} \\ -\frac{K_r K_x}{\kappa _+ - \kappa _-} &{} -\frac{\kappa _-}{\kappa _--\kappa _+} \\ \end{array} \right] \end{aligned}$$

And combining Eq. (37) and the previous matrix definitions we find an exact solution of SUF for an homogeneous root, depending only on the $\mathrm {\frac{k_r}{k_x}}$ ratio. Indeed, by definition:

$$\begin{aligned} SUF_n= \frac{J_{r,n}}{T_{act}} = \frac{K_{r} H_{x,n}}{- \kappa _+ H_{collar} \alpha } \end{aligned}$$

We obtain:

$$\begin{aligned} SUF_n= & {} \frac{K_r}{2 \kappa _+} \left( \left( 1 - \frac{\kappa _+}{K_x} \right) ^n - \left( 1 + \frac{\kappa _+}{K_x} \right) ^n \right) \\&+ \frac{K_r}{2 \kappa _+ \alpha K_x} \left( \left( 1 - \frac{\kappa _+}{K_x} \right) ^n + \left( 1 + \frac{\kappa _+}{K_x} \right) ^n \right) \end{aligned}$$

Let us note that if the segment length is small enough, we can simplify the matrices:

$$\begin{aligned} P\rightarrow & {} \left[ \begin{array}{c c} \frac{1}{\kappa } &{} -\frac{1}{\kappa } \\ 1 &{} 1 \end{array} \right] \\ \varLambda\rightarrow & {} \left[ \begin{array}{c c} 1 +\frac{\kappa }{K_x} &{}0 \\ 0 &{}1 -\frac{\kappa }{K_x} \end{array} \right] \\ P^{-1}\rightarrow & {} \left[ \begin{array}{c c} \frac{\kappa }{2} &{}\frac{1}{2} \\ -\frac{\kappa }{2} &{}\frac{1}{2} \end{array} \right] \end{aligned}$$

And the SUF simply becomes:

$$\begin{aligned} SUF_n \rightarrow -(n+1) \left( \tau l_{seg} \right) ^2 + \frac{\tau l_{seg}}{tanh(\tau l_{root})} \end{aligned}$$

Consequently:

$$\begin{aligned} SUD \rightarrow \frac{\tau }{tanh(\tau l_{root})} \simeq \frac{\tau cosh(\tau z)}{sinh(\tau l_{root})} \end{aligned}$$

the exact solution we found in the continuous model.

## Abbreviations

RWU: Root water uptake
RS: Root system
HA: Hydraulic architecture
SUF: Standard uptake fraction
SUD: Standard uptake density
